# A self-standing three-dimensional covalent organic framework film

**DOI:** 10.1038/s41467-023-35931-4

**Published:** 2023-01-14

**Authors:** Yizhou Yang, Yanyan Chen, Fernando Izquierdo-Ruiz, Clara Schäfer, Martin Rahm, Karl Börjesson

**Affiliations:** 1 Depertment of Chemistry and Molecular Biology, University of Gothenburg, Kemivägen 10, Gothenburg, 41296 Sweden; 2 Department of Life Sciences, Chalmers University of Technology, Kemivägen 10, Gothenburg, 41296 Sweden; 3 Department of Chemistry and Chemical Engineering, Chalmers University of Technology, Kemivägen 10, Gothenburg, 41296 Sweden

**Keywords:** Self-assembly, Organic-inorganic nanostructures, Polymers

## Abstract

Covalent crystals such as diamonds are a class of fascinating materials that are challenging to fabricate in the form of thin films. This is because spatial kinetic control of bond formation is required to create covalently bonded crystal films. Directional crystal growth is commonly achieved by chemical vapor deposition, an approach that is hampered by technical complexity and associated high cost. Here we report on a liquid-liquid interfacial approach based on physical-organic considerations to synthesize an ultrathin covalent crystal film. By distributing reactants into separate phases using hydrophobicity, the chemical reaction is confined to an interface that orients the crystal growth. A molecular-smooth interface combined with in-plane isotropic conditions enables the synthesis of films on a centimeter size scale with a uniform thickness of 13 nm. The film exhibits considerable mechanical robustness enabling a free-standing length of 37 µm, as well as a clearly anisotropic chemical structure and crystal lattice alignment.

## Abstract (web summary)

Fabrication of 3D covalent organic framework thin films is challenging. Here the authors report on a liquid-liquid interfacial approach based on physical-organic considerations to synthesize an ultrathin covalent crystal film.

## Introduction

Covalent crystals, lattice structures maintained by covalent bonds, are fundamentally different from other categories of crystalline materials^1^. Compared to metallic, ionic, or intermolecular interactions, covalent bonds are stronger and more directional^2^. The challenge of making covalent crystals, such as diamond, quartz, and silicon carbide, is often associated with chemical bond formation that is practically irreversible. Reactions therefore must be highly selective to ensure sufficient crystallinity. To make a covalent crystal in a particular shape, for instance, an ultrathin film, chemical reactions need to be selectively constrained along particular directions. A common example and the so far most mature method for manufacturing covalent crystal films is the controlled fabrication of ultrathin diamonds through chemical vapor deposition (CVD), which constrains the crystal growth to the gas-solid interface^3–5^. However, this method is expensive, requires high temperatures, and is not compatible with the production of large-area free-standing (i.e., non-substrate supported) covalent crystal films^6^. In this work, we describe the synthesis of thin and at the same time free-standing covalent crystal films at room temperature without the aid of expensive infrastructure.

Covalent crystals are related to covalent organic frameworks (COFs), which are defined as network-type lattice structures maintained by covalent bonds and based only on light atoms. Such organic framework material can either extend into two or three dimensions^7^. Extensive exploration of new structures^8–10^, topologies^11–13^, methods of fabrication^14–18^, and applications^19–23^ of COFs is motivated by a heightened control of atom and pore distributions as well as the properties that derive therefrom. One successful approach to COF crystal synthesis relies on dynamic covalent chemistry^24–29^. Such chemistry has, for example, lead to the fabrication of single crystals of 2D COFs with a size over 1 µm^25^, and single crystals of 3D COFs with a size reaching tens of µm^26,27^. With films of 2D COFs being realized^30–33^ even with monocrystallinity^34^ using interfacial synthesis methods, 3D COF crystals in film state still remains a challenge. Because 3D COFs are structurally supported by covalent bonds stretching out in three-dimensions, the manufacture of films from them require an extra level of control over the crystal growth.

Here a highly crystalline 3D COF thin film is made by a liquid-liquid interfacial synthesis approach. Due to the controlled distribution of reactants and catalyst in separate phases, the 3D COF formation is selectively confined at the interface between top- and bottom-phases. The confined reaction results in an ultra-low thickness of 13 nm for a covalent crystal. At the same time, films can be produced on a centimeter size scale, giving an aspect ratio of over 10^6^. The films show a high uniformity, a low surface roughness, and are robust enough to sustain a free-standing state with considerable mechanical strength. Due to the low thickness and the free-standing feature, the crystallinity of the 3D COF can be clearly observed under high resolution transmission electron microscopy (TEM), showing well aligned crystal lattice patterns. To summarize, the here discussed method allows free standing covalent crystal films to be made at low cost and at a quality similar to that achieved with the CVD method.

## Results

**Fig. 1:**
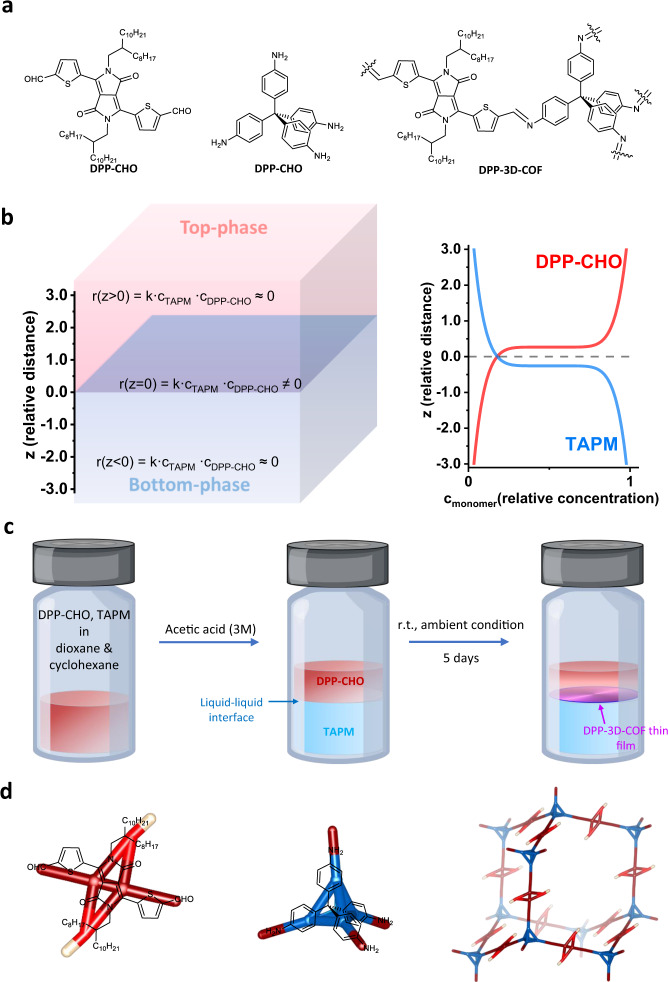
The fabrication of DPP-3D-COF thin films. **a** The chemical structures of the building blocks DPP-CHO and TAPM, and the chemical structure of the DPP-3D-COF. **b** Schematics of the kinetics of the reaction between DPP-CHO and TAPM in the liquid-liquid system. The top phase is a mixture of cyclohexane and dioxane. The bottom phase is a mixture of aqueous acetic acid and dioxane. When equilibrium is reached, the concentration of TAPM in the top phase is close to zero and the concentration of DPP-CHO in the bottom phase is close to zero. **c** Illustration of the liquid-liquid interfacial synthesis method used for fabrication of DPP-3D-COF thin films. The monomers DPP-CHO and TAPM are fully dissolved in a mix of dioxane and cyclohexane. With the addition of aqueous acetic acid, two phases appear after the redistribution of solvents and solutes. The top-phase is colored, while the bottom-phase is colorless, illustrating the distribution of DPP-CHO. After 5 days, a film of the DPP-3D-COF has formed at the interface between the two phases. **d** Illustration of DPP-3D-COF constructed from covalently linked amine tetrapods and aldehyde linkers as molecular sub-units.

For a typical 3D COF, made by an imine condensation reaction, the reaction rate is isotropic under homogenous acidic conditions. As a result, the network stretches out into all three dimensions to build up a crystal bulk rather than a film. Bottom-up synthesis of covalent crystal films, therefore, requires control of the network propagation process with a highly vertically dependent reaction rate. This can be achieved in a phase-separated liquid-liquid system, for instance, formed by mixing cyclohexane, 1,4-dioxane and aqueous acetic acid. In our case we use building blocks of a hydrophobic diketopyrrolopyrrole derivative (DPP-CHO) and a hydrophilic tetrapod-amine (TAPM) (shown in Fig. 1a). These molecules can form imine linkages for the synthesis of DPP-3D-COF. The reactants dissolving-distribution study (Supplementary Fig. 1) reveals that when the system reaches equilibrium, DPP-CHO with its long aliphatic side chains is almost exclusively present in the hydrophobic top-phase, while TAPM is primarily dissolved in the aqueous acetic acid bottom-phase (Supplementary Fig. 2). Accordingly, the reaction rate is close to zero in both top- and bottom-phases but has a finite value at the interfacial plane (Fig. 1b). Thus, the condensation reaction between DPP-CHO and TAPM only occurs in the two-dimensional plane of the interface, which sets a boundary for the DPP-3D-COF network elongation and eventually results in the film state of the material. It is worth pointing out that every site on the interface has the identical reaction rate when the liquid-liquid phase system reaches equilibrium, which is significant for the uniformity of the fabricated film. With reaction progress, a thin film is observed to form at the interface (Fig. 1c), which did not form in control experiments where aniline replaced TAPM in the synthesis (Supplementary Fig. 3). In contrast to the starting material, the obtained DPP-3D-COF film is unable to be dissolved in any of the used solvents (Supplementary Fig. 4), indicating a cross-linked polymeric state (Fig. 1d). After a wash-removal procedure of the top-phase (see experimental section), the DPP-3D-COF film can be transferred to a substrate of choice for further characterization.

### Large area, free-standing film

**Fig. 2:**
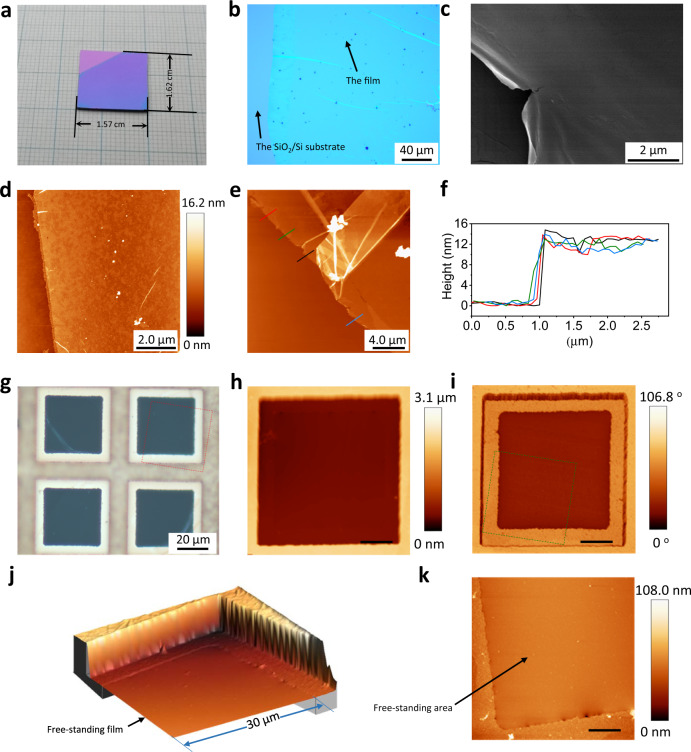
Morphological characterization of DPP-3D-COF films from macroscale to nanoscale. **a** A DPP-3D-COF film on a SiO_2_/Si substrate. The calculated area of the film is 2.5 cm^2^. The top left part is not covered by the film resulting in a different color. **b** Optical image of a film. **c** SEM image of a film showing a manually made rapture in detail. **d** AFM height image of a film that shows uniformity in detail. **e** AFM height image of a film that shows wrinkles. **f** Cross-sectional profiles at the edge of the film. The positions of profile extractions are shown in **e**. **g** A film on copper grids. The top-left and the bottom-right holes have broken films, and the top-right and bottom-left have free-standing intact films. **h**, **i** AFM height-image and corresponding phase-image of free-standing films on copper grids. **j** A 3D image of a film on a copper grid (corresponding to the rectangular area marked by the red dashed line in **g**). **k** AFM height image of the central area in a copper grid showing the inner edge of the grid and the free-standing film (corresponding to the rectangular area marked by the green dash line in **i**).

Following fabrication, DPP-3D-COF films can be transferred onto silicon wafers to observe the macroscopic film integrity and to probe its morphology. Fig. 2a shows a continuous and uniform thin film with a size of over 2.5 cm^2^. Under an optical microscope (Fig. 2b), no discernible holes or breaks can be seen within the observed area (size of ~200 µm) except some wrinkles that are unavoidable for ultra-thin film manipulation, displaying a high integrity on the macroscopic level. Closer examination of the surface conditions was made using Scanning Electron Microscopy (SEM) at different magnifications (Fig. 2c and Supplementary Fig. 5). Under a high (~8 µm) magnification, Fig. 2c shows a DPP-3D-COF film surface without any internal cracks or pinholes on the µm scale. The fine detail of a ruptured edge further illustrates an ultra-low thickness of the film. In a lower magnification (~50 µm) (Supplementary Fig. 5), it can be seen that the inner part has as uniform morphology as in the magnified image of Fig. 2c. Atomic Force Microscopy (AFM) scanning was used for a more quantitative analysis and corroborates a flat surface morphology (Fig. 2d). A statistical analysis of an area of 25 μm^2^ (Supplementary Fig. 6) gives a root mean square (RMS) surface roughness of 0.5 nm that is comparable to the surface of silicon or quartz^35^, which is extremely difficult to reach for a 3D network material. To measure the average film thickness, the height image of a larger area was collected at the edge of the film (Fig. 2e). The cross-sectional profile at four different sites along the edge of the film were extracted and are shown in Fig. 2f. The film thicknesses are around 13 nm, and are very similar at the different positions, indicating a uniform thickness. The constant thickness is derived from the aforementioned equal lateral reaction rate at the liquid-liquid interface (Fig. 1b). Thus, our liquid-liquid interfacial synthesis permits the fabrication of homogenous films with areas up to 2.5 cm^2^, which translates to a six order of magnitude aspect ratio between the thickness and length scale. As a comparison experiment, a film of COF-300 was also synthesized using the same method (Supplementary Figs. 7–9). However, the surface smoothness of the fabricated COF-300 film is seriously deteriorated, which is a result from less confinement of the reaction to the interface. Thus, efficient separation of monomers in different phases contributes to a strict interface reaction and ensures a high quality for the COF film.

To investigate the robustness and potential free-standing nature of such 13 nm thick films, they were placed over 37.46 × 37.46 µm holes on a copper grid (Fig. 2g and Supplementary Fig. 10). A film that is intact over the hollow area has sufficient mechanical strength to make it free-standing. In contrast to the blank copper grids, copper grids holding DPP-3D-COF films exhibit a light brown color in the frame area (Supplementary Fig. 10d, e), indicating the successful loading of films. Under higher magnification (Fig. 2g), films above the hollow area were nearly transparent due to their low thicknesses. Although it is difficult to observe the transparent film directly, the existence of it on hollow sites could be seen by dust particles on an intact area or unevenness from rolled up edges on a broken area (Supplementary Fig. 11). Whereas more than half of the hollow sites were covered by broken films, a considerable amount was covered by perfectly intact pieces (Supplementary Fig. 12). In other words, 13 nm thick films appear robust enough to be able to sustain a free-standing area of ca 1400 µm^2^.

**Fig. 3:**
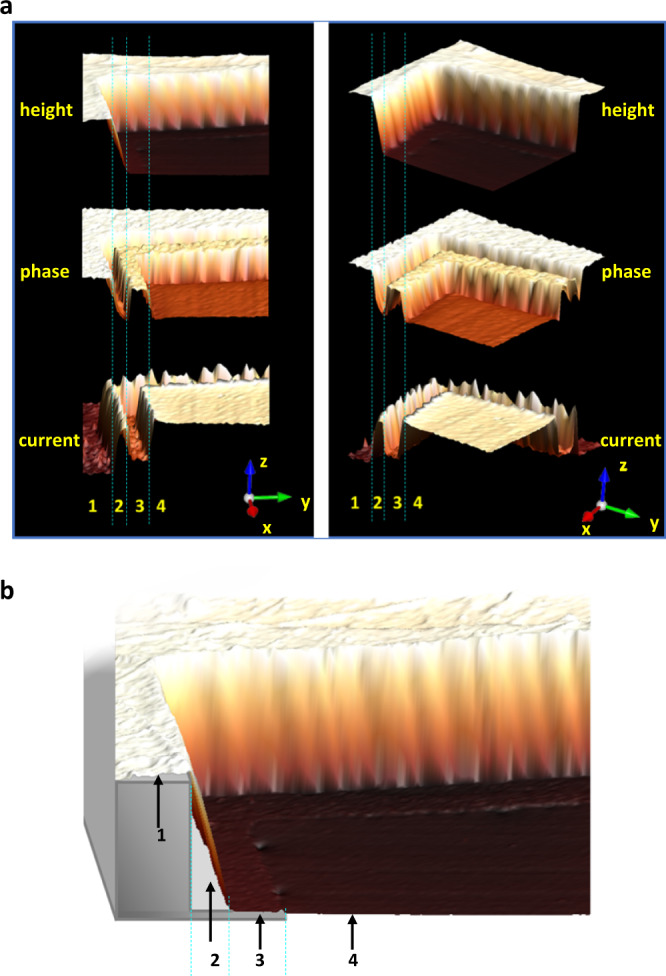
AFM showing details about how a DPP-3D-COF film is held by the Cu grid. **a** AFM height, phase and current 3D images in two different angles (left and right). Area 1 represents the area of the film supported by the frame of the copper grid, 2 represents the area of the film in a tilted free-standing state, 3 represents the area of the film supported by the inner edge of the copper grid, 4 represents the area of the film in a flat free-standing state. **b** AFM height image of the film together with a 3D model of the Cu grid. By comparing the edge-to-edge distance of the Cu frame to the effective distance along the film (as calculated using Euclidean geometry), a strain of 9% was calculated.

Combined measurements from multi-functional AFM (height, phase, and peak force) can provide additional and more accurate information on materials constitution, topography, and mechanical properties like stiffness. The height and the corresponding phase images of an intact free-standing film over one hollow hole is shown in Fig. 2h, i (see Supplementary Fig. 13 for an overview scan). For an ultra-thin film, one could expect the phase to be dependent on what is underneath it. It can be seen that the central area of the hole is roughly at the same height level as the inner edge of the copper grid (Supplementary Fig. 14), showing that the film suspends over the hollow area. Furthermore, the film with and without copper support underneath have a sharp difference in the phase image, supporting a picture of films attached at the inner edges of the copper grid. The sectional 3D image (Fig. 2j) shows how a film is bending down along the inner wall of the copper grid and that the inner edge of the grid is holding the film in place. The film adheres to the top frame and inner edge but is forced to a stretched and tilted free-standing state between the top frame and inner edge with curvatures at the junctures (Fig. 3, area 2). The abrupt drop from the top frame to the lower edge that holds the film in place forces the film to strain about 9%. Such elasticity is uncommon for covalently bonded 3D networks. The absence of rupture under these stretched conditions reflects a considerable robustness of the film. At the same time, it is striking to observe that a fully covalently bonded 3D framework material can behave with some softness and flexibility when in an ultra-thin state.

**Fig. 4:**
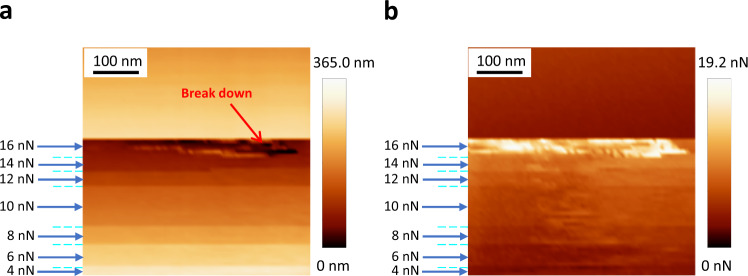
Indentation punching test of a DPP-3F-COF free-standing film by AFM peak force QNM technology. A step-increasing force was applied on the tip (radius lower than 12 nm) from 4 nN to 16 nN. The AFM height image is shown as **a** and the corresponding adhesion image is shown as **b**. Puncture was caused under a force of 16 nN.

Quantitative nanomechanical (QNM) properties were measured as a Young’s modulus mapping by peak force function AFM (Supplementary Fig. 15)^36^. The observed flexibility is corroborated by a modulus of 95.6 MPa, which is notably lower than reported 3D COFs^29,37^. This means that DPP-3D-COF is a soft COF, which is extraordinary for a material with the same covalently formed topology as diamond. This value is on par with the lower end of observations made on organic crystals^38^ and is in the vicinity of elastomers^39^. A selective AFM scan within the hollow hole (the green square in Fig. 2i) shown in Fig. 2k and Supplementary Fig. 16, shows that the free-standing area of the film is considerably flat. No bending down towards a central point can be seen, which probably reflects a stretched film of a quite high mechanical strength. For a quantitative presentation of the robustness, AFM peak force QNM technology was used to conduct indentation tests on the free-standing area with gradually increased peak force (Fig. 4). The film remained intact until it was punctured at a force of 16 nN, which is close to the limitation of the used cantilever. As the peak force increased, the indentation depth increased (Fig. 4a). However, when a scan with constant peak force was afterward performed (Supplementary Fig. 17a, b), the film showed the same surface height over the whole area, which indicate that the film returned to its original shape and is therefore elastic. Furthermore, a constant 16 nN force scan creates a hole in the film rather than entire film collapse (Supplementary Fig. 17c, d). In summary, the aspect ratio between the free-standing length (37.46 μm) and thickness (13 nm) of the film is 2.9 × 10^3^, and it shows considerable flexibility and mechanical robustness.

### Chemical structure confirmation

**Fig. 5:**
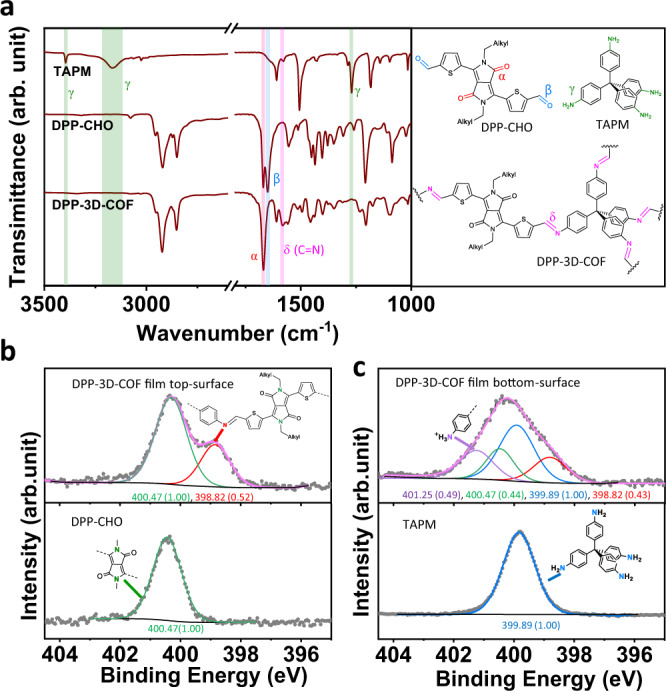
FTIR and XPS spectra of DPP-CHO, TAPM, and DPP−3D-COF. **a** FTIR of the starting materials and the prepared DPP−3D-COF film as well as chemical structures. The key comparisons between functional groups and corresponding peaks in the FTIR spectra is color coded. **b** Comparison of the N 1 *s* XPS spectra of the top surface of the DPP−3D-COF film and the starting material DPP-CHO. The inserted chemical structures show the connection between *N* atom varieties and peaks. For the top-surface of the film, the ratio of peak areas between the green and red peak is 1.00/0.52 = 1.9. Green lines are fits to N in DPP-CHO, red line is a fit to N in imine linkage, pink line is a sum of fits to the whole spectrum. **c** The comparison of N*1s* XPS spectra of the bottom surface of the DPP−3D-COF film and the starting material TAPM. The inserted chemical structures show the connection between N atom varieties and peaks. For the bottom-surface of the film, the ratio of peak areas between purple + blue peaks and the red peak is (0.49 + 1.00)/0.43 = 3.5. Blue lines are fits to *N* in TAPM; purple line is a fit to protonated amine in TAMP.

To verify the chemical integrity of DPP-3D-COF films, FTIR, Raman and X-ray (XPS) photoelectron spectroscopy were used to systematically investigate the presence of relevant functional groups. Fig. 5a shows the FTIR of reactants (DPP-CHO and TAPM) as well as the product (DPP-3D-COF). By comparing reactants and product, evidence for the condensation reaction between the amine and the aldehyde to form an imine linkage can be clearly seen. Generally, the FTIR spectrum of DPP3D-COF is a combination of the DPP-CHO and TAPM spectra. However, some peaks ascribed to functional groups taking part in the reaction have disappeared, and a peak representing the formed imine bond has appeared. The disappearance of N-H stretching vibrations at 3395 and 3166 cm^−1^ in the DPP-3D-COF indicate that the amount of unreacted amine functionalities in the COF is below the detection limit^40^. The absence of reactants is further evidenced by the disappearance of the γ absorption of the C-N stretching at 1269 cm^−1^, which is not active in the DPP-3D-COF due to symmetry^40^. The DPP-CHO reactant has two strong IR absorptions, at 1669 (α) and 1649 cm^−1^ (β), which can be ascribed to the C = O stretching vibrations of the ketone and aldehyde, respectively^41^. In the DPP-3D-COF, the β absorption has disappeared and the typical δ absorption of the C = N stretching of a thiophene 2-position-imine has emerged at 1583 cm^−1^, indicating a high conversion of the aldehyde into an imine linkage^41^. The formation of the C = N imine bond is also confirmed by Raman spectroscopy (Supplementary Fig. 18).

Even with the chemical transformation confirmed, FTIR is based on the transmission through the material, which means it collects the average structural information of the whole material. A 3D COF is theoretically an infinite network extending in space, but all crystals have boundaries, and this is especially true for thin films. It is therefore also relevant to study the chemical structure of the film surfaces. Knowledge of surface compositions can provide insights into details of the interfacial reaction. XPS measures binding energies of atoms and is a suitable method for investigating the chemical surface structure of films. High resolution XPS spectra of the top surface (Fig. 5b) shows two peaks, at 400.47 eV and 398.82 eV, corresponding to the 1 s levels of nitrogen in the DPP unit and the newly formed C = N imine linkage, respectively^42–44^. The ratio of the two peaks is 1.9:1, which is higher than the theoretical ratio of 1:1 for a perfect infinite DPP-3D-COF network. Instead, the observed ratio is close to the 2:1 ratio expected for a surface completely covered with imine-bonded DPP-CHO (Supplementary Figs. 19a and 20) This indicates an excessive amount of DPP residual units exposed on the top surface. In comparison, the bottom surface of the film shows a different N *1* *s* spectrum (Fig. 5c). Besides the 1 s N peaks of the DPP unit core and the imine linkages, there are two additional peaks, located at 401.25 eV and 399.89 eV, which we attribute to the N of ammonium, –NH_3_^+^, and an amine group, -NH_2_^45,46^, respectively. The atom ratio between N of the imine and the combined N of ammonium and amines is 1:3.5. The latter ratio approaches that of 1/4 reacted TAPM. Put together, our XPS studies imply that the bottom surface of the COF film is primarily terminated by TAPM (Supplementary Fig. 19b). Interestingly, the surface terminating mode of the film can be changed when the respective top/bottom surfaces are exposed to the reversed phase (Supplementary Fig. 21). Additionally, the S *2p* peak of DPP-3D-COF is similar to DPP-CHO and without any peak appearing at 169 eV (Supplementary Fig. 22), indicating that the thiophene moieties were not oxidized during COF formation^47^. A combination of IR spectroscopy and XPS show that imine linkages between tetrapods and linear units extend throughout the film, which ends with DPP-CHO on the top surface, and TAPM on the bottom surface. It is worth mentioning that the liquid-liquid environment during the synthesis of the film plays a vital role for the product. The rate limiting step is related to concentration, and the water soluble TAPM caps the bottom surface, and the hydrophobic DPP-CHO caps the top surface. This results in a detectable anisotropy of the chemical structure in the different facets.

### The crystallinity of films

**Fig. 6:**
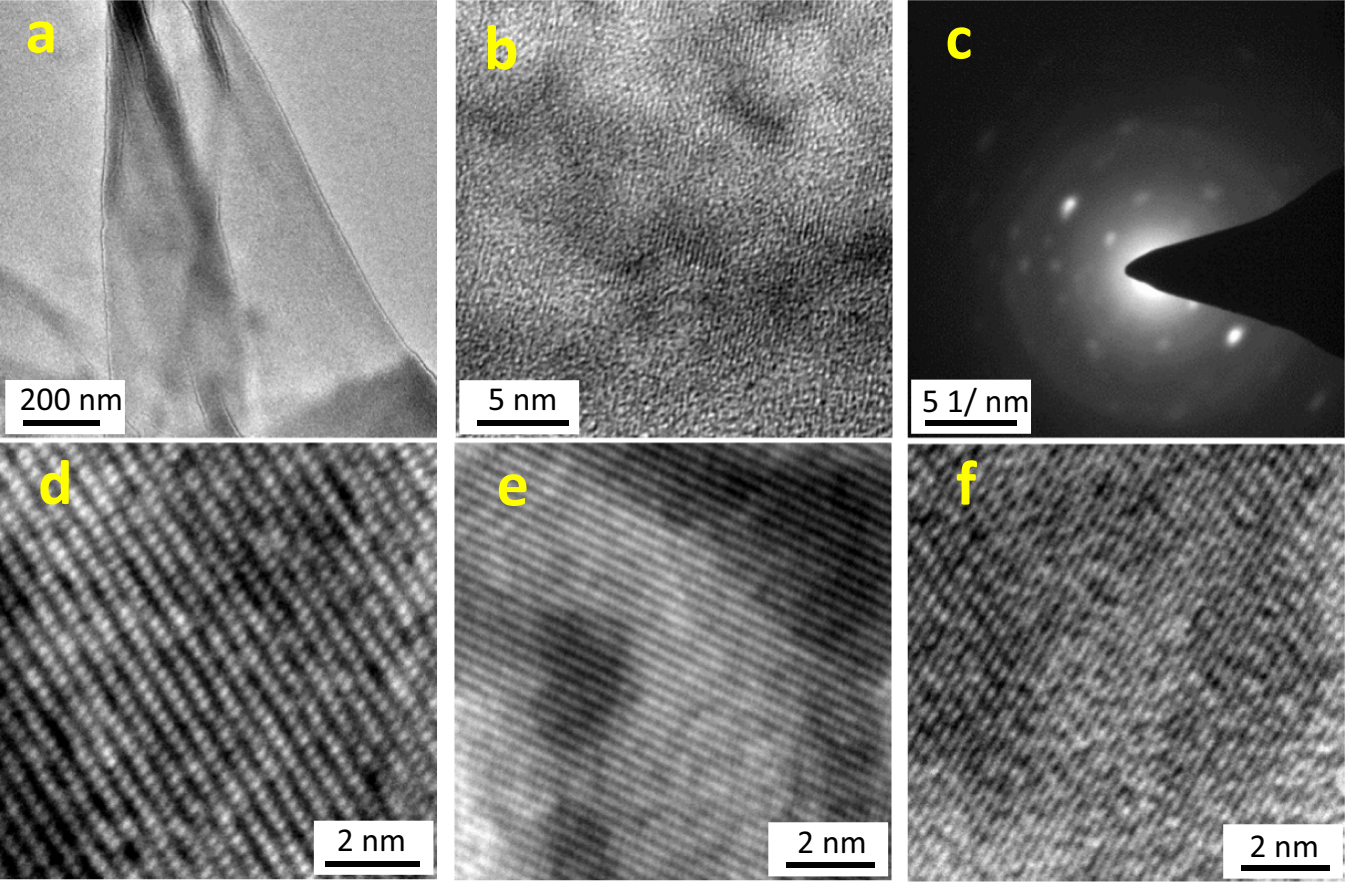
Transmission electron microscopy (TEM) characterization of DPP-3D-COF films. **a** Large scale TEM image of a DPP-3D-film. The film was fabricated via interfacial synthesis and transferred to a copper grid for characterization. **b** TEM image at higher magnification of the film shown in **a**. The lattice pattern all over the visual field indicate a high crystallinity. **c** Selected area electron diffraction pattern (SAED) of the same thin film, further corroborating the high crystallinity. **d–f** High resolution TEM of films, showing different crystal lattice orientations.

Having evaluated the morphology, mechanical property, and the chemical structure of the DPP-3D-COF film, we next turn our attention to its crystallinity. Due to the ultra-thin state of the film, electrons can penetrate through it and the crystallinity is therefore best observed by transmission electron microscopy (TEM). As shown in Fig. 6a, large areas of the DPP-3D-COF film have a uniform and light shade, indicating high electron transmission and thus low thickness. Furthermore, some areas are darker and appears to be wrinkled. When observed under higher magnification (Fig. 6b), the sites of the film within the focus plane contain noticeable lattice patterns having the same orientation, clearly illustrating the crystalline state of the film. Selected area electron diffraction (SAED) was collected as shown in Fig. 6c. The SAED exhibits an unambiguous dot pattern, indicating that the observed area is single-crystalline. To evaluate the domain size, the full width at half maximum (FWHM) of the main electron diffraction was extracted, which gives a crystallite correlation length of 27.0 nm through a Scherrer grain size analysis (Supplementary Fig. 23). This value is expected to be considerable affected by the thinness of the film, and can therefore be regarded as a lower estimate in the lateral direction. A High-resolution TEM image (Fig. 6d–f) show details on the 2D projection of the 3D structure in one orientation. HRTEM images were captured at several areas of films from different batches to obtain representative lattice features of different projection angles. Two types of patterns, dot arrays and densely stacked lines, dominate in the projections of the 3D networks (Fig. 6d–f). Particularly, the highly ordered voids in the 3D framework structures can be clearly observed in Fig. 6d. These well-aligned lattice structures in different orientations are clear evidence that the DPP-3D-COF film is a covalent crystalline film.

Notably, all the measured d-spacing of lattices (Supplementary Fig. 24) are lower than the simulated unit cell parameters of a non-penetrated diamond structure (Supplementary Fig. 25, Supplementary Table 1). This discrepancy hints to the existence of interpenetration in the COF film, which is very hard to avoid in 3D COFs unless using specific strategies^48,49^. Further, the measured d-spacing distance of the lattice was found to fit well with a five-fold interpenetrated diamond structure (***dia-c5***, Supplementary Fig. 26, Supplementary Table 2). In fact, the dot array pattern with d-spacing of 4.6 Å (Supplementary Fig. 24a) matches with the distance of the orthogonally distributed voids from the dia-c5 network. The other two stacked linear patterns were also matched with projections of the five-fold interpenetrated network from different orientations (Supplementary Fig. 24b, c), further supporting a five-fold interpenetrated network. The observed short distance between lattice planes is a result of the five-fold interpenetration of networks. It is worth noting that the bulky flexible side chains on the DPP unit cannot be neglected when assessing the physical properties of the film. After all, the alkyl chain is associated with a large volume: the atom count is C_40_H_82_ per chain, compared to the corresponding framework fragment atom count of C_28.5_H_20_N_4_O_2_S_2_. Thus, it is reasonable to assume a very limited porosity for the DPP-3D-COF film due to the bulky alkyl side chains and the interpenetration phenomenon.

Grazing incidence x-ray diffraction (GIXRD) was utilized to further investigate the crystallinity. However, as expected from the 13 nm-thickness, the quantity of a single-film sample is too small to gain sufficient signal to noise ratio. In order for an acceptable quality of data to be collected, batches of films were gathered on the substrate for the measurement until the diffractions were clearly seen. As shown in Supplementary Fig. 27, there are three noticeable peaks located at 3.83°, 5.29°, and 20.8°, illustrating the crystallinity of the DPP-3D-COF film. The observed diffractions match well with a simulated XRD spectrum based on a five-fold interpenetrated DPP-3D-COF structure, further corroborating an interpenetrated state of the prepared COF film. The broadness of observed peaks is caused by the small crystal domain size resulted from the low thickness of the film, which sets a limitation of 6 repeating layers in the framework. It is worth mentioning that the crystallinity of the DPP-3D-COF was not affected after treatment to different solvents (Supplementary Fig. 28).

We have synthesized covalent crystal films using a liquid-liquid interfacial synthesis approach. Despite having a thickness of only ~13 nm, films can cover a macroscopic area (2.5 cm^2^) with few defects and low surface roughness. Constructed using strong covalent linkages between well-defined molecular building blocks, the ultra-thin films show considerable mechanical robustness that can endure a free-standing area of 37 x 37 µm. Also, films were observed to be stretched by ~9%. Elasticity is a quite uncommon phenomenon among fully-covalently bonded structures, and we hypothesize that the low thickness in conjunction with a considerable side chain flexibility to be the reason for this observation. Extensive characterization reveals highly ordered interconnected and interpenetrated 3D-covalent organic framework films with an area-to-thickness aspect ratio of six orders of magnitude. The high mechanical strength of films allows for a free-standing length to aspect ratio of 2.9 × 10^3^ and it enabled us to investigate the boundaries of a covalent crystal. The DPP-3D-COF network is terminated by different building blocks on the top and bottom surfaces, resulting in a chemically anisotropic film. This research opens a way to fabricate covalent crystal films. The crystallinity of the films can reach the level of those done by chemical vapor deposition; however, the liquid-liquid approach directs the synthesis of organic materials and requires no expensive equipment. We hypothesize that one of the main features of the liquid-liquid interface is to provide a molecularly flat template and controlled reaction kinetics for film growth. We hope that this research will provide inspiration and facilitate the development of this fascinating field, especially toward covalent crystal membranes.

## Methods

### Chemicals and materials

All chemicals were purchased from commercial suppliers and used without further purification, including TAPM (>95%, TCI), glacial acetic acid (100%, VWR), 1, 4-dioxane (99.8%, Sigma-Aldrich), cyclohexane (99.5%, Sigma-Aldrich). The copper grids used for holding free-standing film were purchased from SPI Supplies. DPP-CHO monomer was synthesized according to the literature reported procedure^50^.

### DPP-3D-COF film fabrication

1.5 ml DPP-CHO solution (1.9 × 10^−3 ^M, in 1, 4-dioxane), 1.5 ml TAPM solution (1.3 × 10^−3 ^M, in 1, 4-dioxane) and 1.0 ml cyclohexane were mixed together in a vial (20 ml volume, 2 cm diameter) to form a red uniform solution. Then 0.8 ml aqueous acetic acid (3 M) was added and the vial was capped. After shaking the vial for 5 s, the system was settled statically to reach the solution redistribution balance among the two liquid phases. The bottom phase is colorless and the top phase is deep red. The system was placed in a vibration free environment under ambient conditions for 24 h. Then, another 4 ml acetic acid (3 M) was added. The vial was capped and placed in vibration free environment. After 5 days, the thin film could be observed at the interface of the two phases. To get rid of unreacted monomers, a wash-removal procedure was used. 2 ml cyclohexane was drop-added and removed from the top phase very gently without causing any interruption of the interface. The wash-removal procedure was repeated until the top phase changed from red to completely colorless. Finally, the clear thin film was obtained at the interface and could be transferred to wanted substrates.

### COF-300 film fabrication

1.5 ml Terephthalaldehyde (TPA) solution (5.6 × 10^−3 ^M, in 1, 4-dioxane), 1.5 ml TAPM solution (3.7 × 10^−3 ^M, in 1, 4-dioxane) and 1.0 ml cyclohexane were mixed together in a vial (20 ml volume, 2 cm diameter) to form a red uniform solution. Then 0.8 ml aqueous acetic acid (3 M) was added and the vial was capped. After shaking the vial for 5 s, the system was settled statically to reach the solution redistribution balance among the two liquid phases. The system was placed in a vibration free environment under ambient conditions for 24 h. Then, another 4 ml acetic acid (3 M) was added. The vial was capped and placed in vibration free environment. After 5 days, the thin film could be observed at the interface of the two phases.

### Instrumentations

The UV-vis spectra were measured on a Lambda 950 UV/VIS/NIR Spectrometer from PerkinElmer. A Zeiss Axioscope 5 was used to observe the image of films in reflective mode. 1H NMR spectra were recorded on a Bruker 700 Avance III spectrometer equipped with a 5 mm QCI cryoprobe (700 MHz ^1^H; 176 MHz ^13^C) using CDCl_3_, containing 0.03% tetramethylsilane with 0.00 ppm as an internal reference or using dmso-d6 as solvent, as solvent. SEM was conducted using a JEOL JSM-6301F scanning electron microscope with an acceleration voltage of 12 kV. The SEM image was performed on three different batches of films. An NT-MDT NTEGRA AFM was used for surface morphology measurements, including height, phase and current images. The TAP−150Al-G Silicon Probes for AFM measurements was purchased from Budget Sensors. The morphology was measured and collected more than 10 times in order to confirm the reproducibility. Peakforce QNM characterization was conducted on an SPM-Bruker Dimension ICON with ScanAsyst cantilevers. Indentaion test was repeated 3 times and showed reproducible results. FTIR spectra were measured and repeated on a Bruker Invenio R instrument in transmission mode. Raman spectra were measured using a WITec alpha300 R Raman Microscope equipped with a 532 nm laser and were reproducible in-between different batches of films. The XPS analysis was performed on an ESCALab 250Xi (Thermo Scientific) using 200 W monochromatized Al Kα radiation. To avoid deviation among different areas, at least three different spots were randomly measured. TEM was carried out using a FEI Tecnai T20 transmission electron microscope with a LaB6 electron source under the acceleration voltage of 200 kV. Different batches of films were observed with clear lattices. Grazing incidence WAXS data were obtained on a Mat:Nordic SAXSLAB instrument. The detector used was a Pilatus3 300 K R from Dectris, and the source was a Rigaku Micromax −003 with a Cu target. The wavelength is 1.54 Å. The spectra are similar for over 5 measured batches.

### Computer simulations

The tetrahedral structure of TAPM was modeled without symmetry constraints (P1 symmetry) using a primitive unit cell inspired by diamond. Alkyl sidechains were replaced by methyl groups to reduce computational costs, resulting in a unit cell composed by 234 atoms. Such large systems are at the limit for what is computationally practical to calculate using Density Functional Theory. To efficiently evaluate these structures, we relied on semi-empirical Density Functional Tight Binding (DFTB) simulations. Calculations were performed using DFTB+, version 21.1^51^. The *mio-1-1* parametrization set^52^ designed for organic molecules was used together with H-damping corrections^53^. Forces were converged within 0.01 eV/Å. The cell was relaxed allowing all the degrees of freedom to be optimized, allowing for deformation out of the initial cubic cell shape of an ideal diamond lattice.

## Supplementary information

Supplementary Information

## Data Availability

The data that support the findings of this work are available within the Article and its Supplementary Information files. Additional data are available from the corresponding authors upon request.
