# Supplementary material for: A self-standing three-dimensional covalent organic framework film
Source: Nat Commun. 2023 Jan 14;14:220. doi: 10.1038/s41467-023-35931-4 (PMC9839775; doi:10.1038/s41467-023-35931-4)
Supplement: Supplementary file 1 — Supplementary Information [file 41467_2023_35931_MOESM1_ESM.pdf]

Supplementary Information:

A self-standing three-dimensional covalent organic framework film

Yizhou Yang[1], Yanyan Chen[2], Fernando Izquierdo-Ruiz[3], Clara Schäfer[1], Martin Rahm[3], Karl Börjesson[1]✉

[1]Depertment of Chemistry and Molecular Biology, University of Gothenburg, Kemivägen 10, Gothenburg 41296, Sweden

[2]Department of Life Sciences, Chalmers University of Technology, Kemivägen 10, Gothenburg 41296, Sweden

[3]Department of Chemistry and Chemical Engineering, Chalmers University of Technology, Kemivägen 10, Gothenburg 41296, Sweden

✉Email: karl.borjesson@gu.se

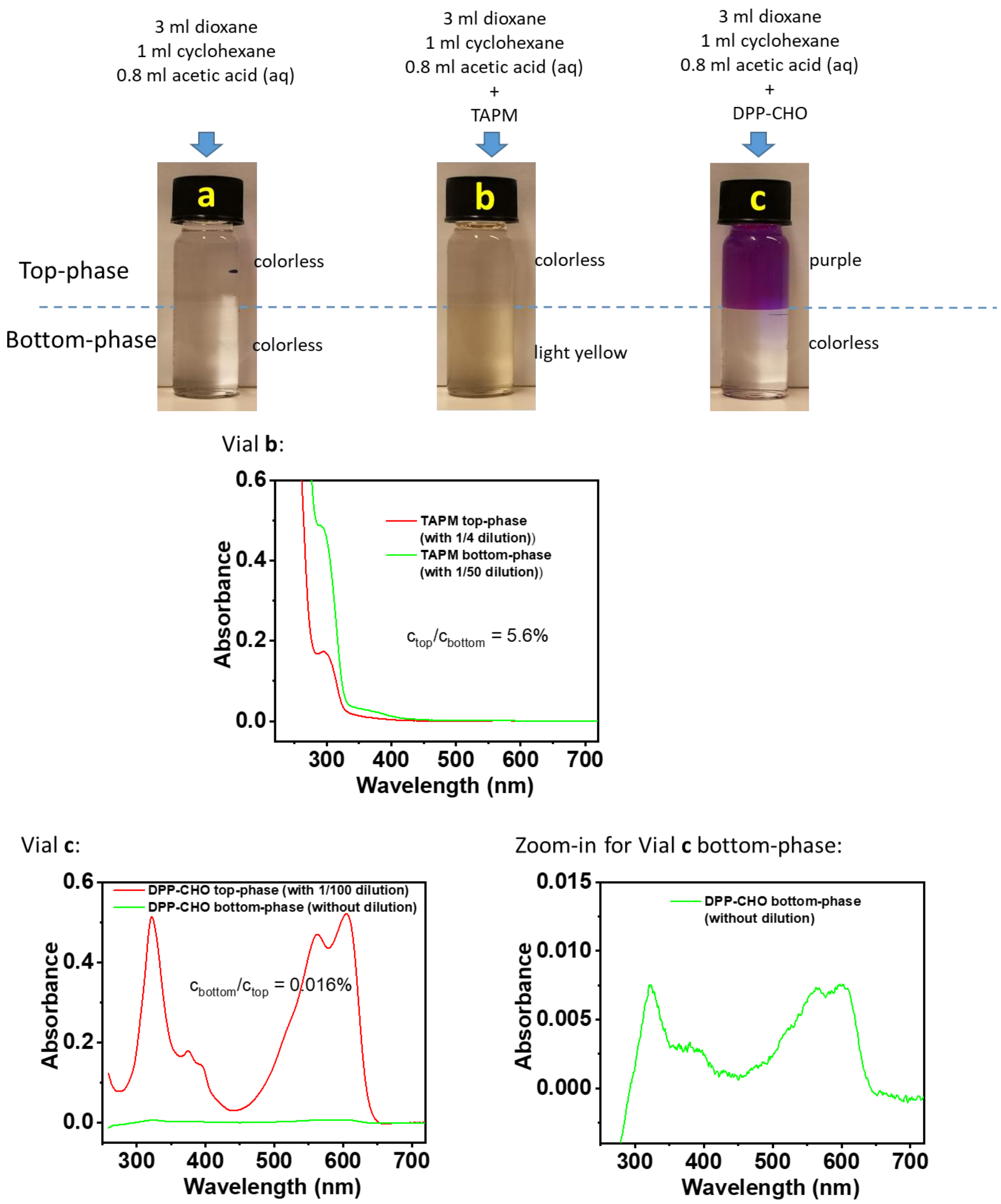

Supplementary Fig. 1| The study of reactant distributions in the liquid-liquid system. To explore how the reactants distribute in a solution containing dioxane, cyclohexane and acetic acid (aq), TAPM and DPP-CHO were dissolved into the solution. The vial **a** is filled with 3 ml dioxane, 1 ml cyclohexane and 0.8 ml acetic acid (aq, 3M), which is the same composition as in the liquid-liquid interfacial synthesis. Both the top and bottom phases are colorless and transparent, and the interface is marked by a blue dash line. 0.75 mg TAPM was dissolved in vial **b**, and 2.7 mg DPP-CHO was dissolved in vial **c**. As shown in vial **b**, the bottom phase shows a light yellow color (TAPM) and the top phase is colorless. In vial **c**, the top phase shows a purple color (DPP-CHO), and the bottom phase is colorless. By measuring the uv-vis absorption of top-/bottom-phase in vial **b**, the concentration of TAPM in bottom phase is known 18 times of TAPM in top phase. By measuring the uv-vis absorption of top-/bottom-phase in vial **c**, the concentration of DPP-CHO in top-phase is known 6250 times of DPP-CHO in bottom-phase.

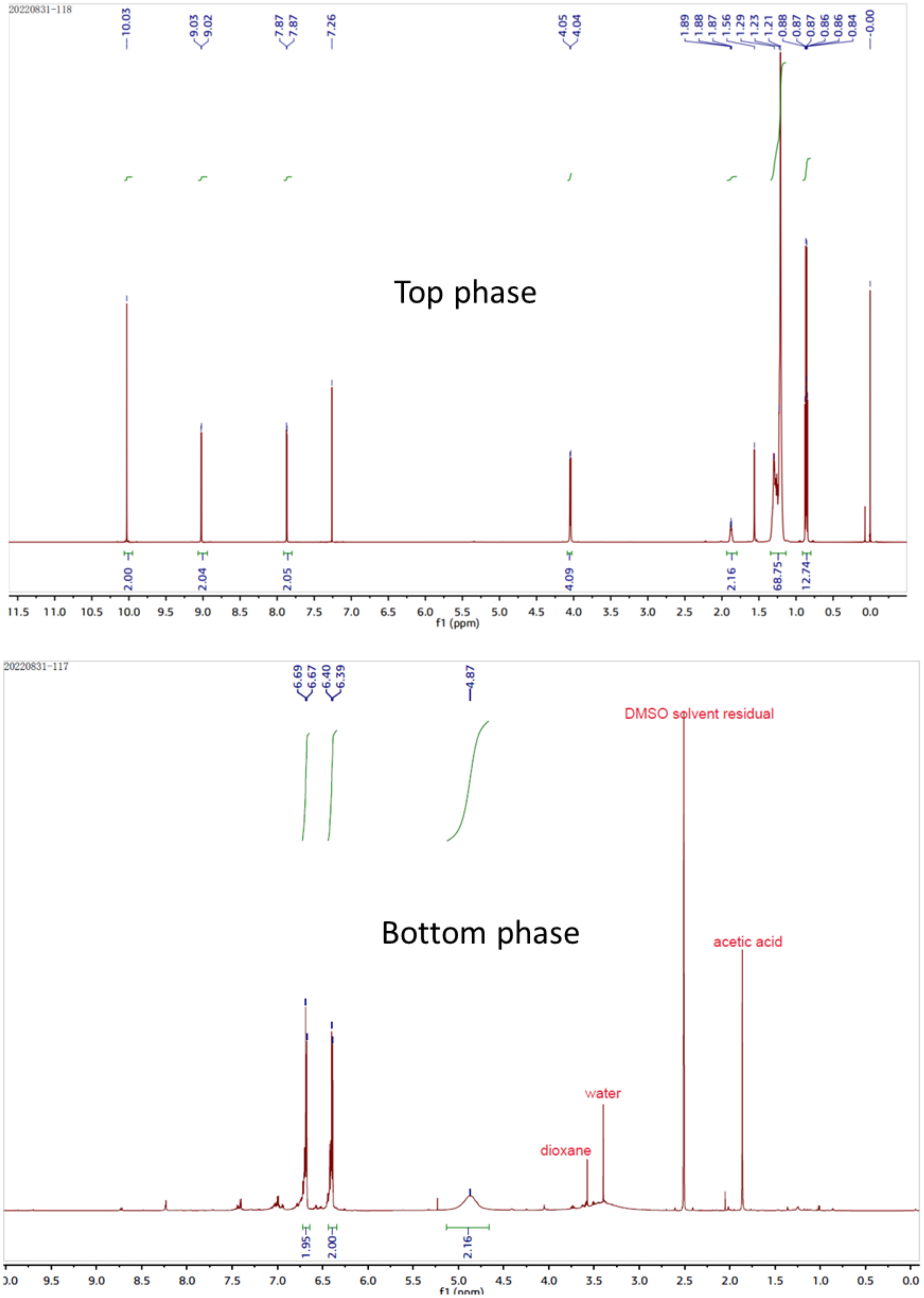

Supplementary Fig. 2| NMR spectra materials in top phase and bottom phase. The NMR spectrum (in $CDCl_3$) from top phase exclusively shows peaks of DPP-CHO monomer. The NMR spectrum (in dmso-d6) from the bottom phase shows the dominate presence of TAPM, besides used solvent and acid for the reaction.

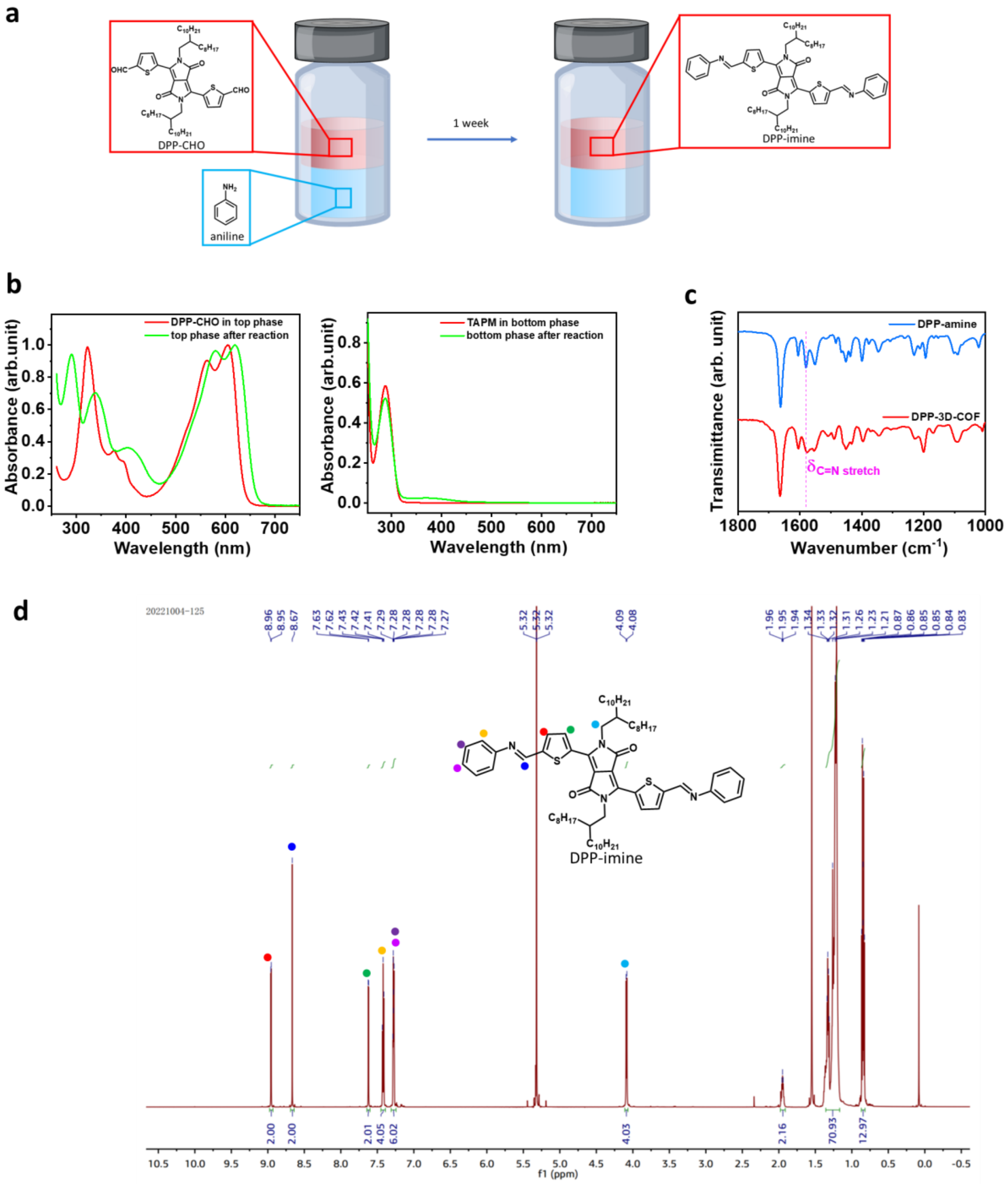

Supplementary Fig. 3| Homogenous imine product synthesis in the liquid-liquid system. In the synthesis, TAPM was replaced by aniline and the reaction condition was otherwise kept the same. The product DPP-imine was found only to exist in the top phase. **a**, Illustration of the reaction system. **b**, Compound distribution before and after reaction in the system studied by Uv-vis spectroscopy. **c**, Comparison of the FTIR of the homogenous product DPP-imine with DPP-3D-COF. **d**, ^{1}H NMR spectrum of the DPP-imine produced in the top phase of the system (CD_2Cl_2 as solvent).

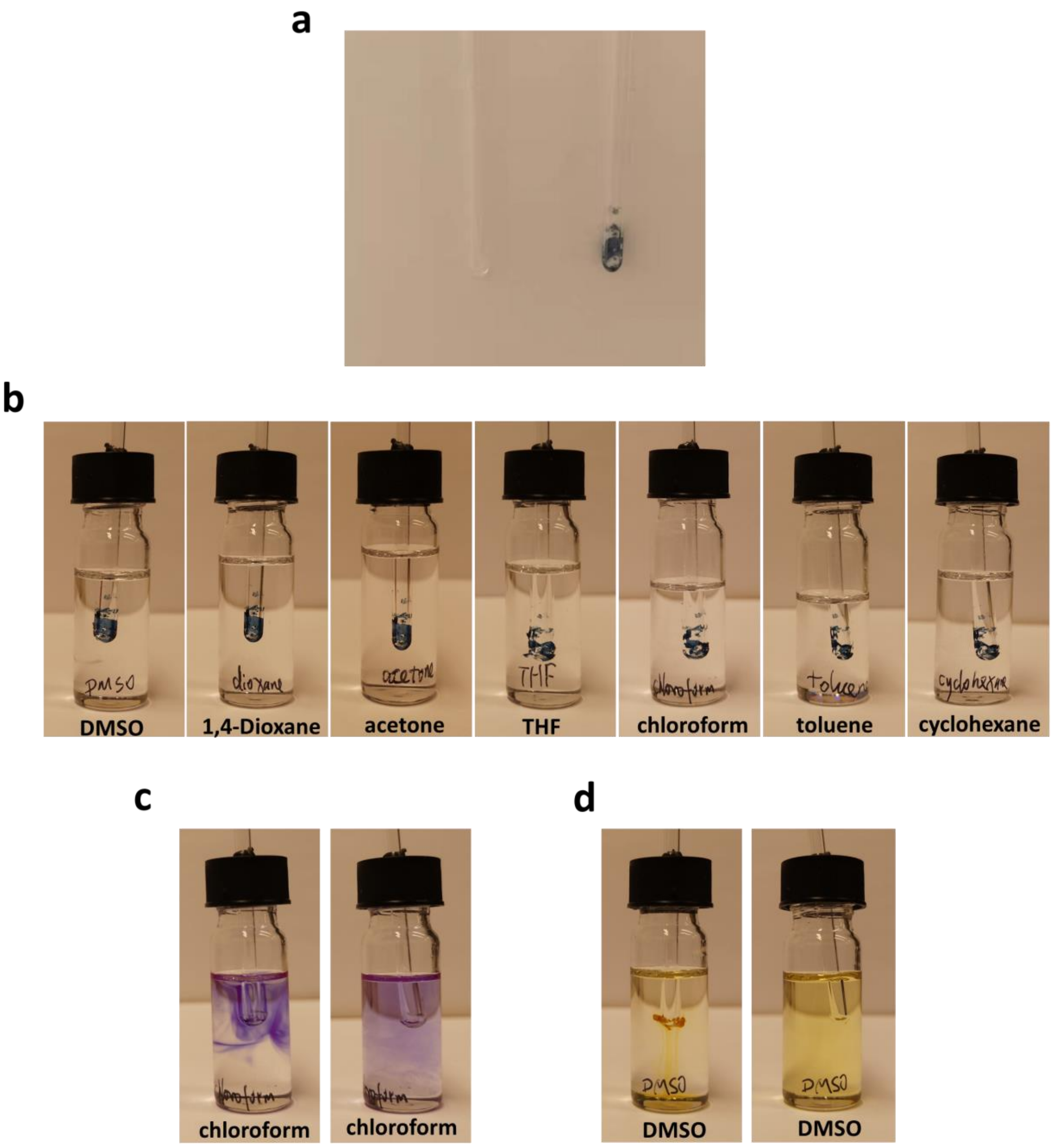

Supplementary Fig. 4| Dissolution test of a DPP-3D-COF film. a, A blank glass tube (left) and a glass tube enwrapped by DPP-3D-COF films from 10 batches (right). **b**, The tube enwrapped with the DPP-3D-COF films in a series of solvents. The photo was captured 30 min after immersion in each kind of solvent. No dissolution was observed as indicated by the solvents being colorless after immersion. It should be mentioned that the different appearance of the film in different solvents were a result from solvent soaking caused deformation and exfoliation of the film from the glass tube. **c**, Glass tube enwrapped with DPP-CHO after being immersed in chloroform for 10 s (left) and 5 min (right). **d**, Glass tube enwrapped with TAPM after being immersed in DMSO for 10 s (left) and 5 min (right).

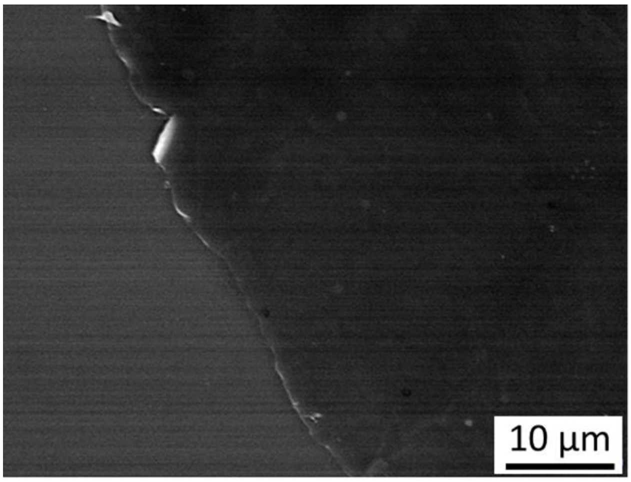

Supplementary Fig. 5| SEM image of DPP-3D-COF thin film showing a high smoothness on a large scale. The DPP-3D-COF film was prepared via the liquid-liquid interfacial synthesis and transferred onto a wafer for SEM imaging. The image was captured near the edge of a large area of the DPP-3D-COF film (with size of at least 1 cm × 1 cm).

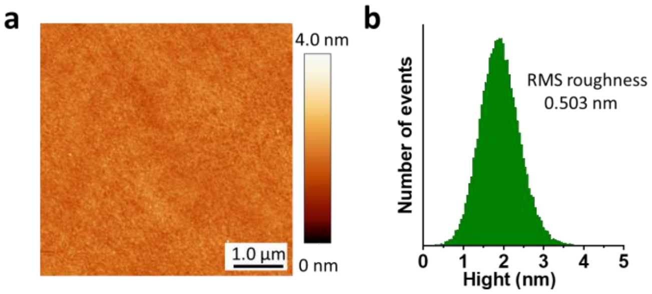

Supplementary Fig. 6| Surface roughness analysis of DPP-3D-COF film. a, AFM height image of a DPP-3D-COF film. **b**, Statistics of the surface height for the image in **a**, giving an RMS roughness of 0.503 nm.

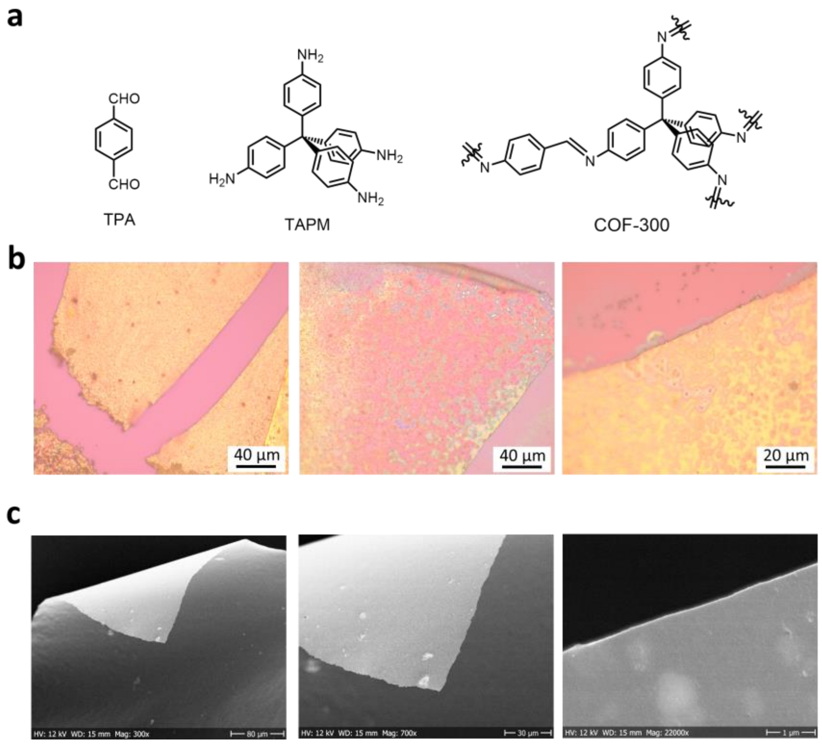

Supplementary Fig. 7| Fabrication of COF-300 films through liquid-liquid interfacial synthesis. a, Chemical structures of monomers used for COF-300 film synthesis and chemical structure of COF-300. **b**, Optical microscope image and **c**, SEM image of COF-300 films.

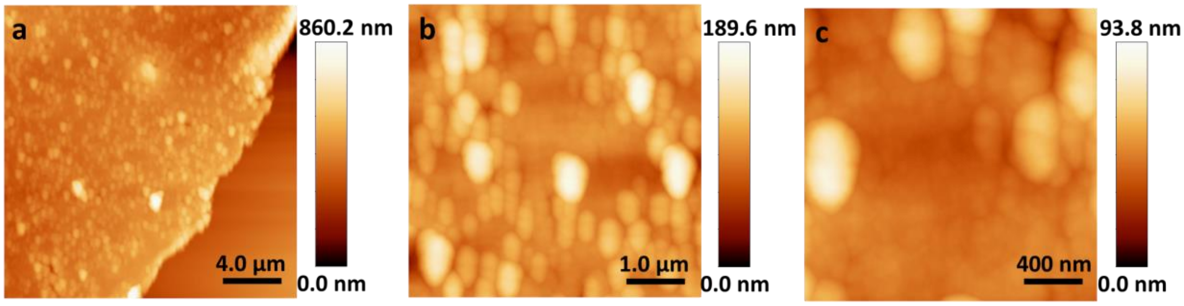

Supplementary Fig. 8| AFM height images of COF-300 films prepared by liquid-liquid interfacial synthesis in different scales. a, AFM scan at the edge of a COF-300 film in large scale (20 μm × 20 μm). **b**, The AFM zoom-in scan (5 μm × 5 μm) at the inner area of image **a**. **c**, AFM further zoom-in scan (2 μm × 2 μm) at the central area of image **b**.

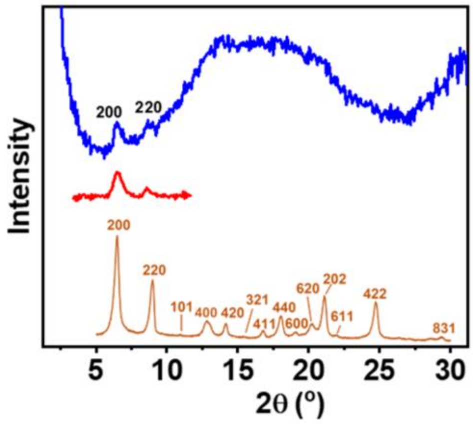

Supplementary Fig. 9| Comparison of GIXRD of COF-300 films made from liquid-liquid interfacial synthesis with reported PXRD of COF-300. Blue line shows the XRD spectrum of a COF-300 film measured in wide-angle scattering (WAXS) mode. The red line shows the XRD spectrum of a COF-300 film measured in mid-angle scattering (MAXS) mode, giving a high signal noise ratio. The pink spectrum shows the literature PXRD of COF-300 reported in literature. Reprinted (adapted) with permission from Supplementary reference 1. Copyright 2018, American Chemical Society.

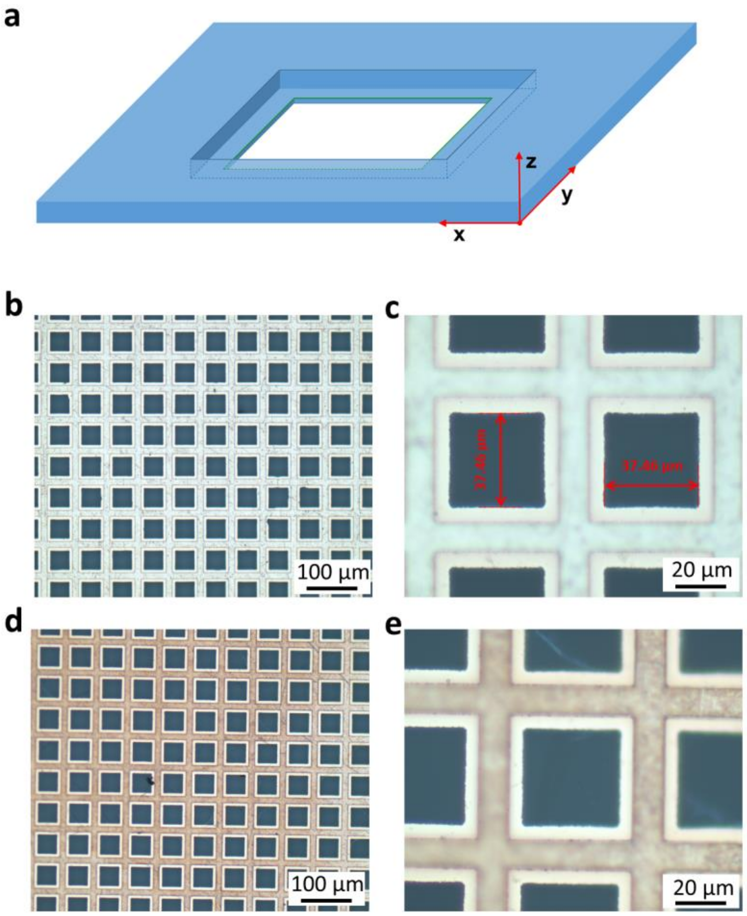

Supplementary Fig. 10| Free-standing DPP-3D-COF film held by Cu grids. a, Shape illustration of the square hollow hole structure of the Cu grid. **b-c,** Optical microscopic image of a blank Cu grid. **d-e,** Optical microscopic image of Cu grids covered by a DPP-3D-COF film.

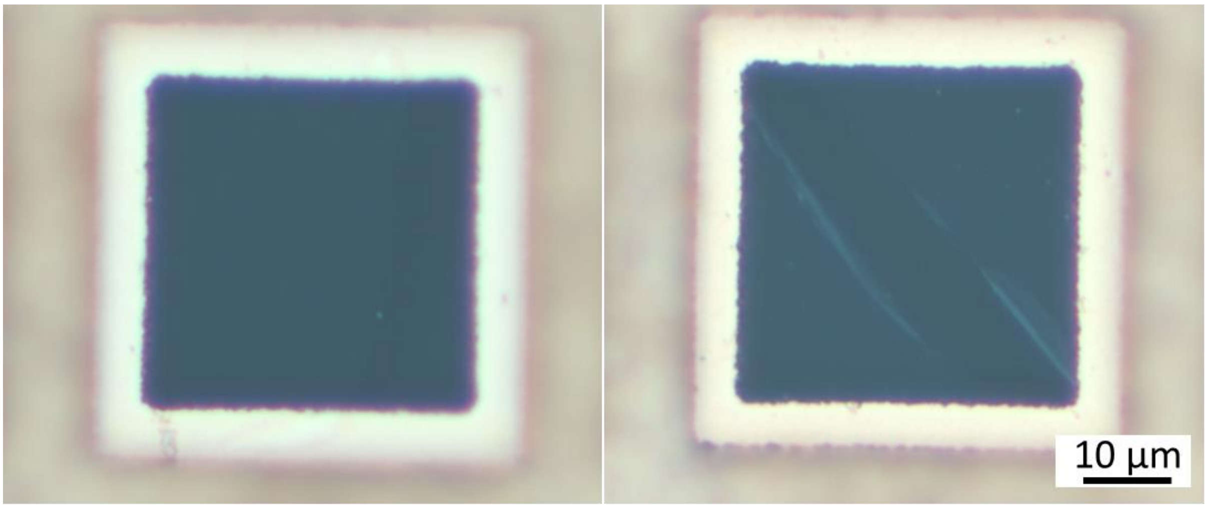

Supplementary Fig. 11| Comparison of an intact free-standing DPP-3D-COF film (left) and a broken DPP-3D-COF film (right) on a Cu grid by optical microscopy. The film is generally transparent and hard to see when a background is lacking. However, a dust particle in the left image indicates the existence of the film. In the right-side image, the film is easier to see due to a roll of film at a tear.

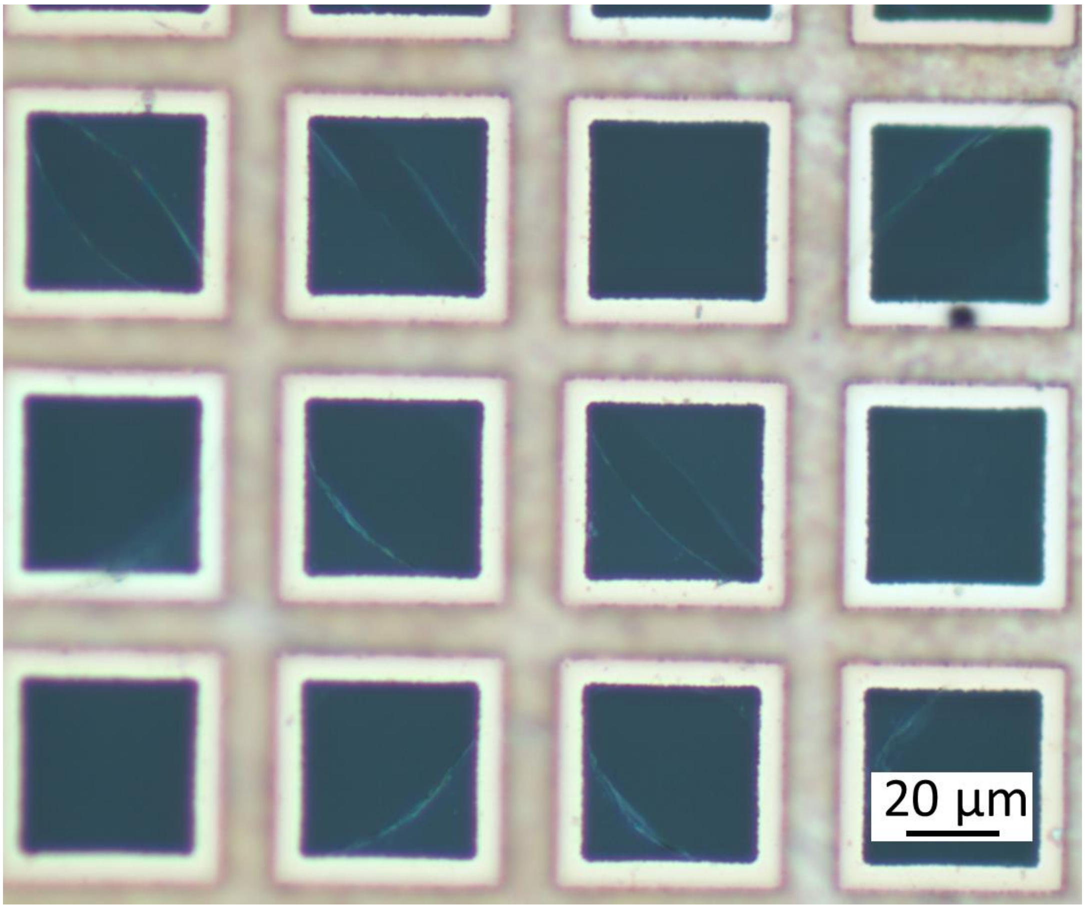

Supplementary Fig. 12| Cu grids holding 12 DPP-3D-COF films captured by optical microscopy. Due to the ultra-thinness of the DPP-3D-COF film, over half of the hollow holes are covered with differently broken films instead of intact free-standing films. However, 4 out of the 12 hollow holes are covered with intact films.

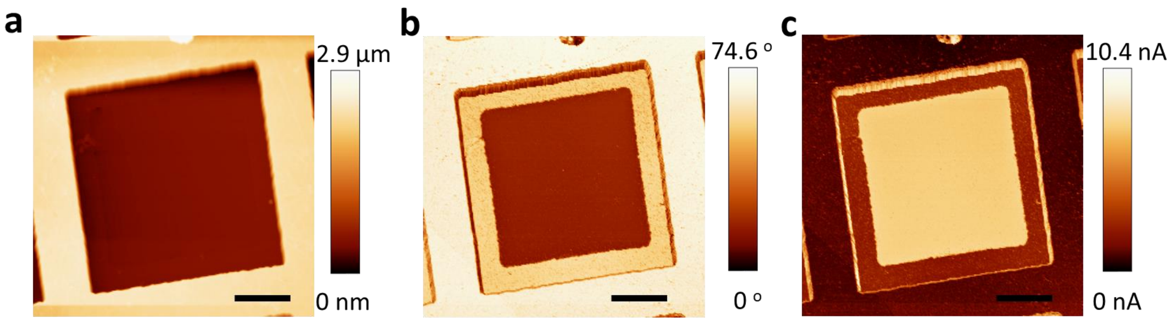

Supplementary Fig. 13| AFM characterization of free-standing films on Cu grids. a, AFM height image showing the height distribution over the scanned area, **b,** AFM phase image showing the phase-shift mapping of the scanned area. Apparently, the film supported by copper grids and film in free-standing state have totally different phase shift, illustrating different film stiffnesses of respective areas, **c,** AFM current image showing the compensation current of the feedback loop, indicating the current change when the AFM tip scan over the whole surface.

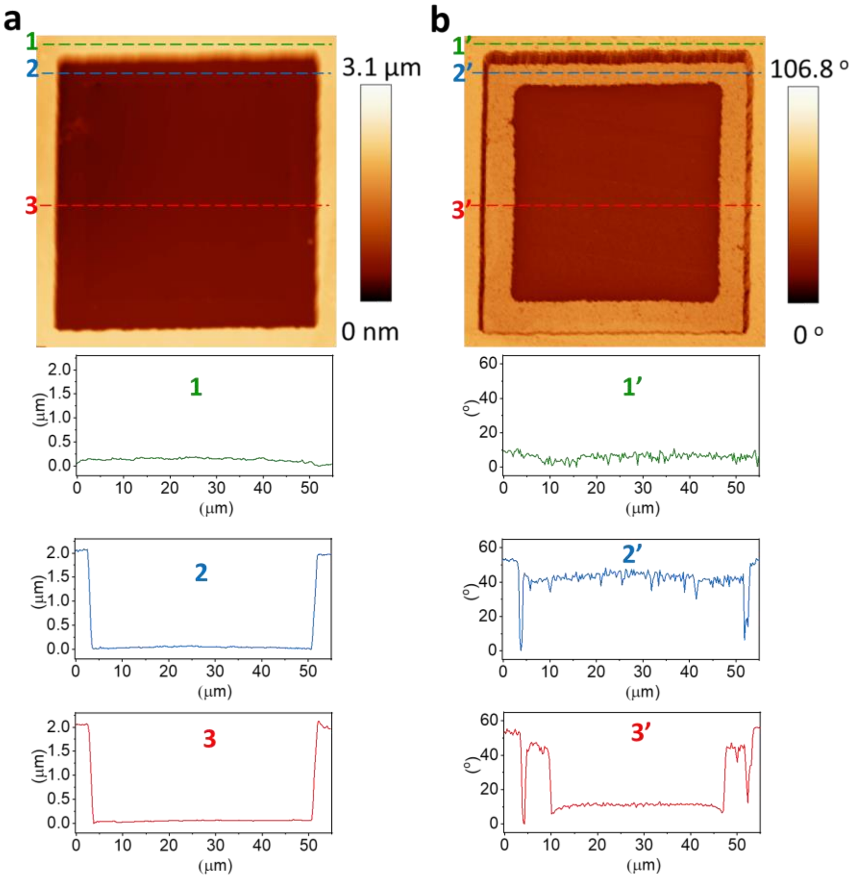

Supplementary Fig. 14| Sectional profiles of an AFM height image and the corresponding phase image extracted at different places on the film on the Cu grid. a, AFM height image of the DPP-3D-COF film on the Cu grids, with sectional profiles extracted 1) across the frame area, 2) across the inner edge area, and 3) across the hollow hole area. **b,** AFM phase image corresponding to **a**, with sectional profiles extracted 1') across the frame area, 2') across the inner edge area, and 3') across the hollow hole area.

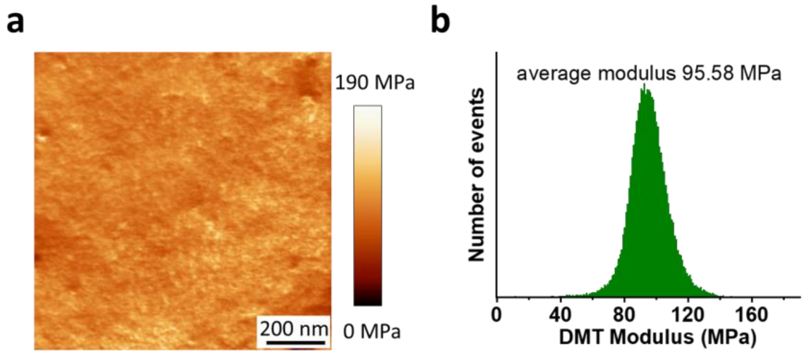

Supplementary Fig. 15| Peak force quantitative nanomechanical (QNM) properties of the DPP-3D-COF film measured by AFM. a, DMT modulus mapping of the film. **b**, Statistics of DMT modulus for the scanned area of a.

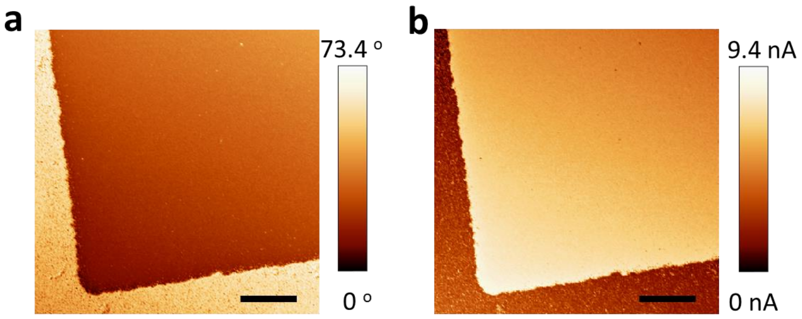

Supplementary Fig. 16| AFM phase image and current image of central area in the copper grids showing the inner edge of Cu grids and the free-standing film, corresponding to the height image displayed in Fig. 2k. a and **b** are phase and current images, respectively. The area of film supported by the inner edge of the grid and the area where the film is free-standing is in totally different phase states, indicating a different mechanical stiffness. Apparently, the ultra-thin film on the Cu surface is much harder than the free-standing film without any support. Also, the AFM cantilever monitored different current between the tip and the film surface in the two different areas.

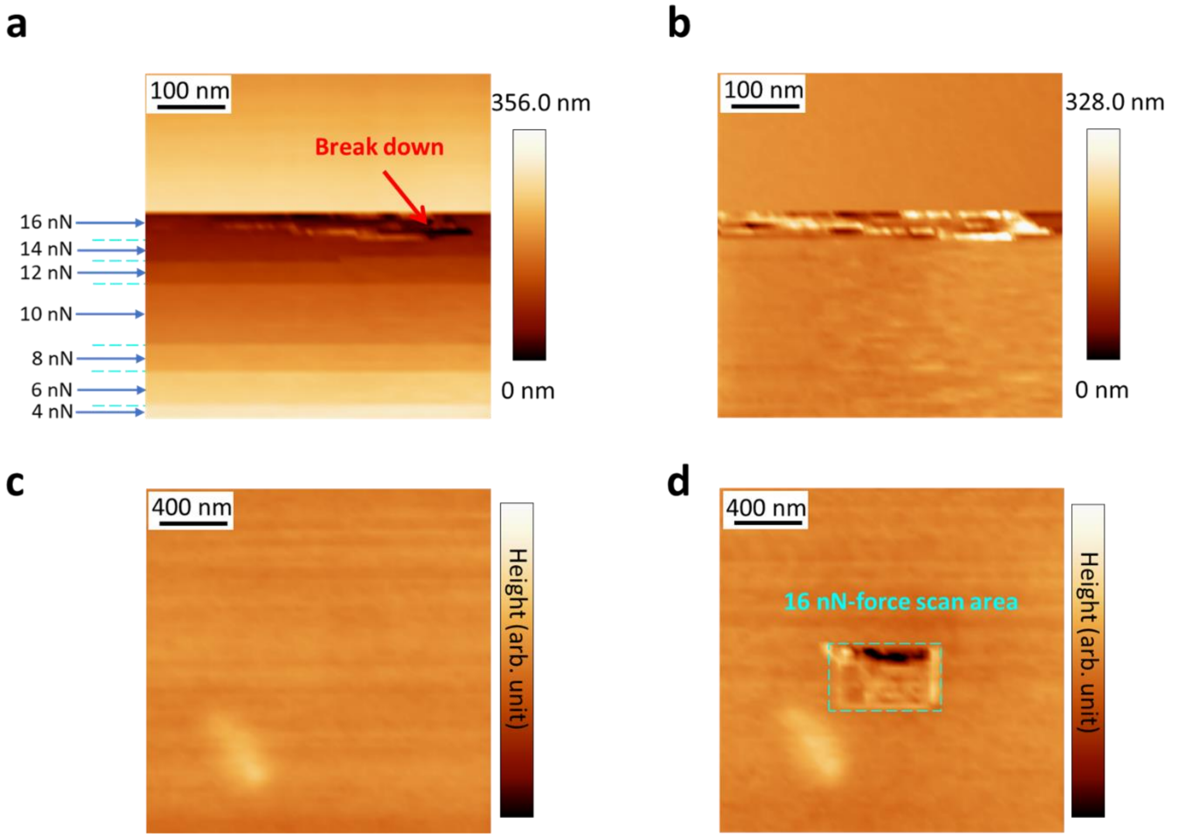

Supplementary Fig. 17| Indentation recovery and presentation of film breakdown after scan by 16 nN force. a, AFM height scan with a step-increasing force applied on the tip (radius lower than 12 nm) from 4 nN to 16 nN (same image as Fig. 4a), showing an increased indentation of the film with applied peak force. **b**, AFM height scan directly after the scan in a, with a constant force applied on the tip, showing a constant height of the film, and thus shape recovery of the film. **c**, The AFM height image of free-standing film before 16 nN punching scan. **d**, The AFM height image of the same site after 16 nN peak force scan. The scanned area is shown in blue dash square.

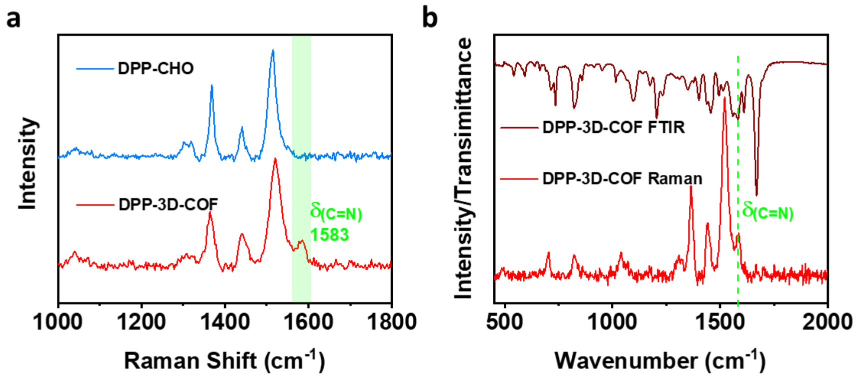

Supplementary Fig. 18| Raman spectra of DPP-CHO and DPP-3D-COF. a, Comparison of Raman spectra of the DPP-3D-COF and the DPP-CHO monomer. The new peak at 1538 corresponds to a C=N stretching vibration, indicating the formation of imine bonds. **b,** Comparison between Raman and FTIR spectra of the DPP-3D-COF. The peaks for the C=N bond are marked by a green dashed line.

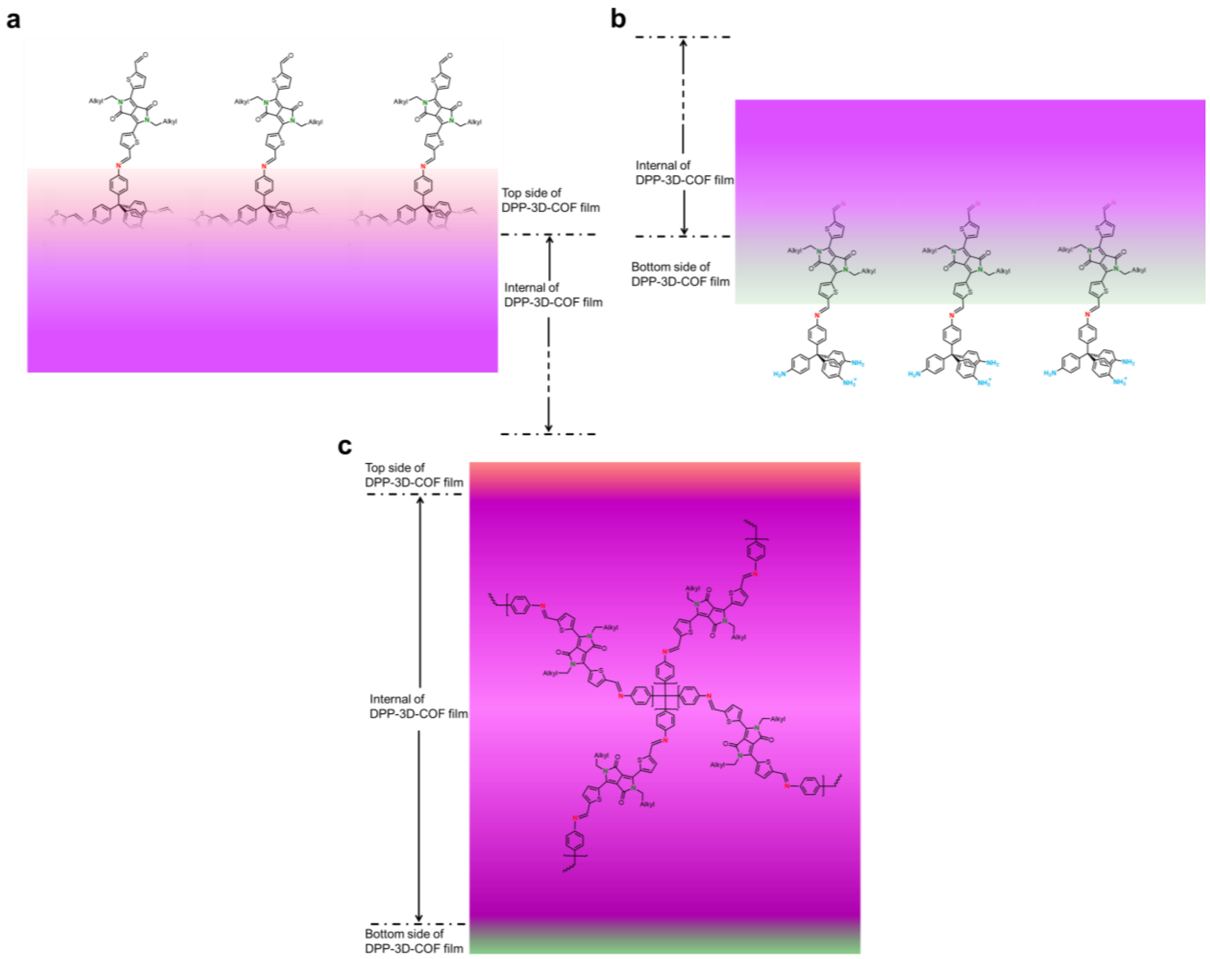

Supplementary Fig. 19| Different chemical states on the top and bottom surfaces, and intererior of the DPP-3D-COF film. a, Chemical state of the top surface, showing the DPP-3D-COF terminating with the imine bonded DPP-CHO building block. **b,** Chemical state of the bottom surface, showing the DPP-3D-COF terminating with the imine bonded TAPM building block. **c,** Chemical state of the interior of the film, showing the ratio of atoms in an infinite extending network.

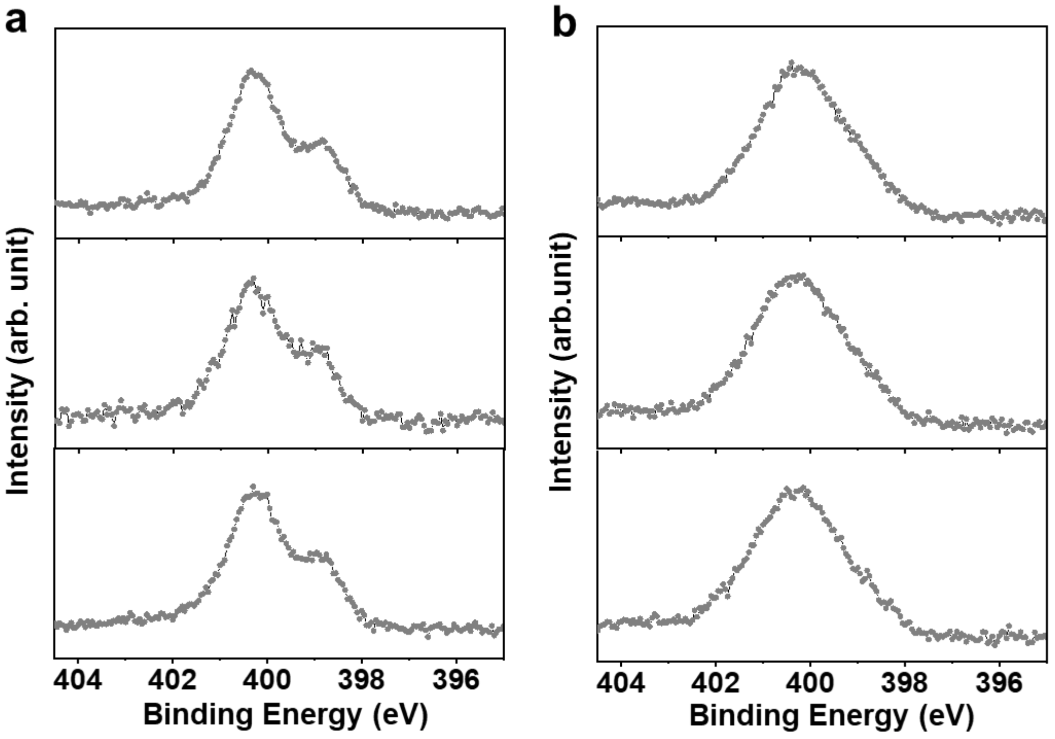

Supplementary Fig. 20| XPS survey for chemical uniformity check on top and bottom surfaces of the DPP-3D-COF film. a, N *1s* XPS spectra measured on three randomly chosen spots on the top surface of the DPP-3D-COF film. **b,** N *1s* XPS spectra measured on three randomly chosen spots on the bottom surface of the DPP-3D-COF film. The spectra are similar to each other within a, as well as within b, indicating that the top/bottom surfaces are uniform in chemical distribution. The figures on top of **a** and **b** are used for deconvolution in Fig. 3b for further analysis.

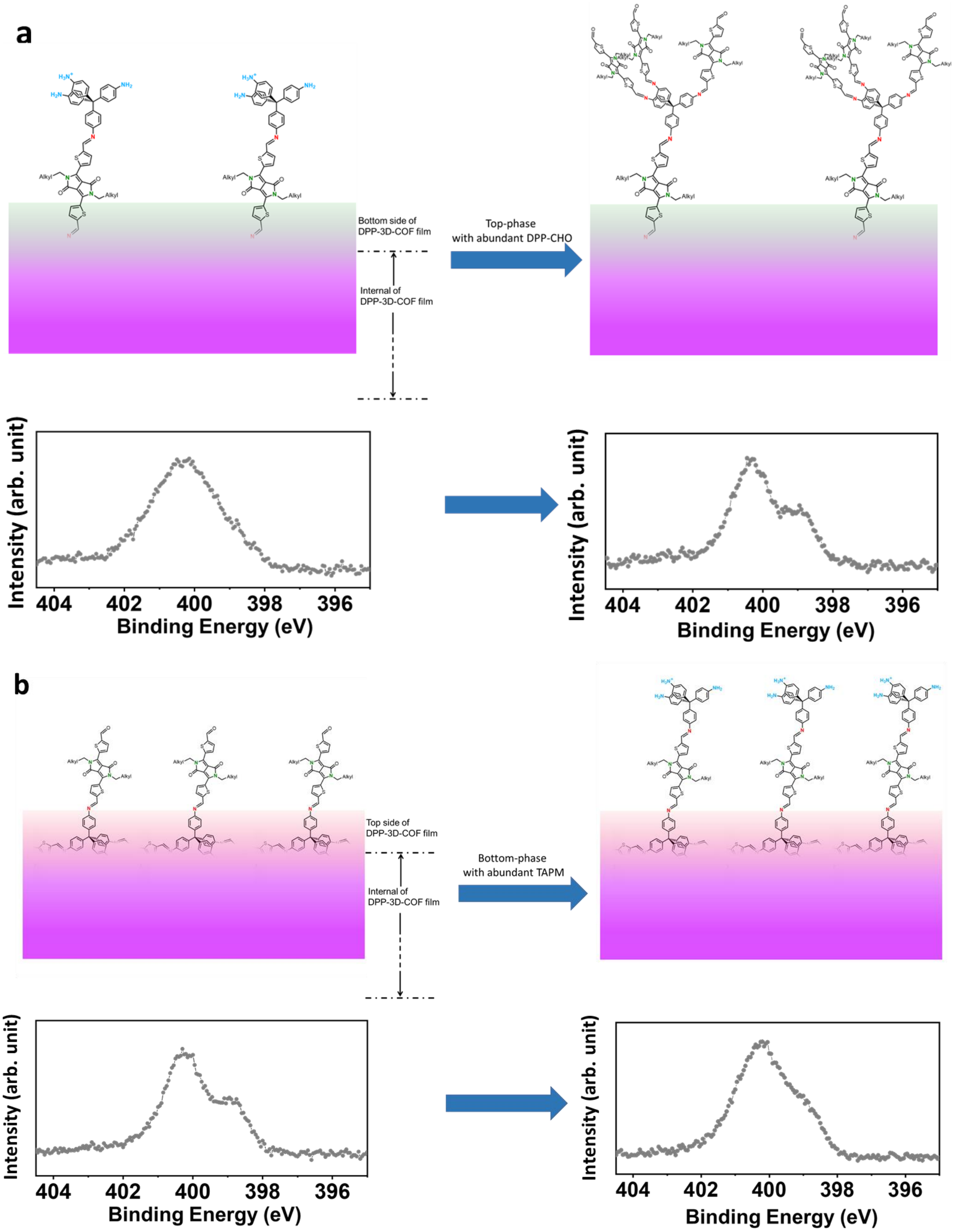

Supplementary Fig. 21| Surface terminating group conversion experiments. a, The N *1s* XPS spectrum of the bottom side after submerged into the top phase solution showed a distinct rise of a peak at 398.8 eV, indicating that the surface is now terminated by DPP-CHO. **b**, The N *1s* XPS spectrum of the top side after submerged into the bottom phase solution showed a weakening of peaks at 398.8 eV and rising of intensity at 401.25 eV. This indicates that the top surface now is mostly terminated by the TAPM monomer, although with a minor DPP-CHO residual.

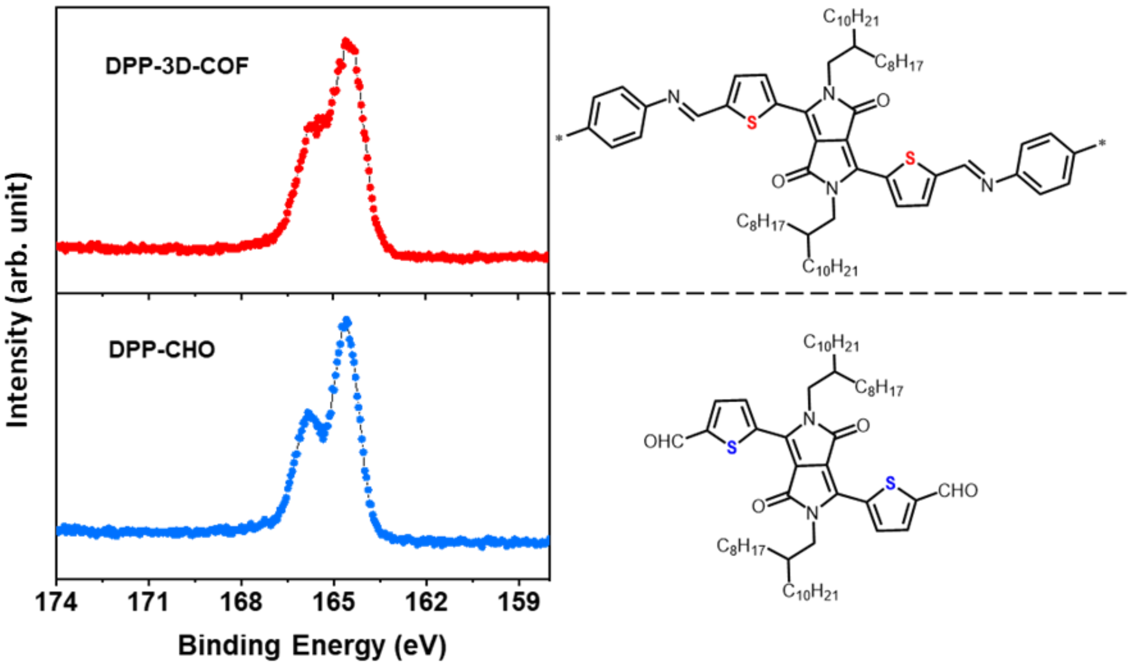

Supplementary Fig22| S *2p* XPS spectra of DPP-3D-COF film (top) and DPP-CHO monomer (bottom). The spectra are similar to each other, indicating the S element stays in the same chemical state. If the thiophene functional group is oxidized during COF formation, there would be a new peak arising at 169 eV.

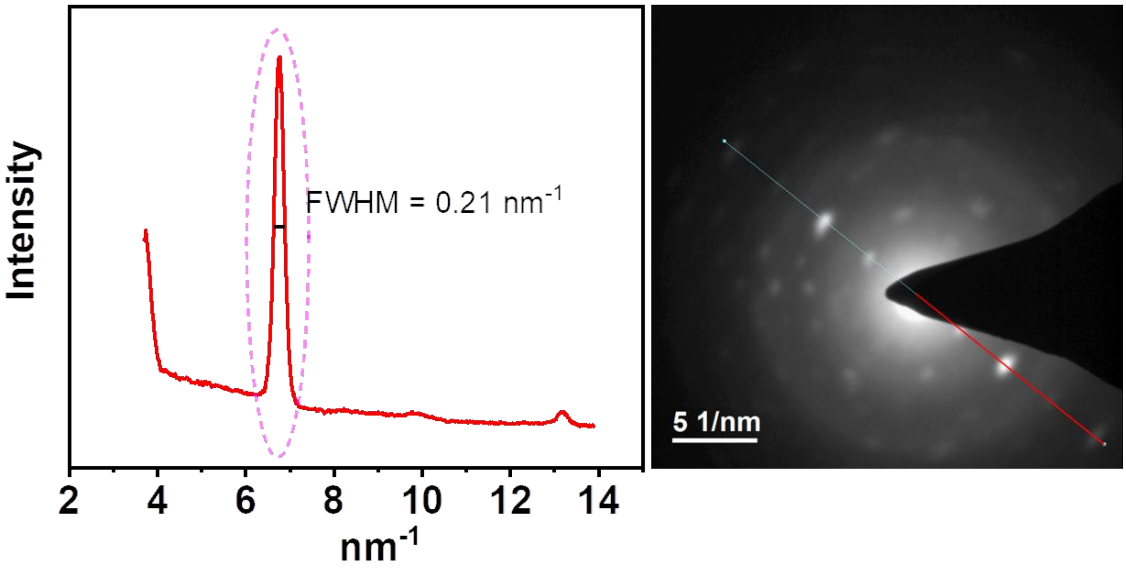

Supplementary Fig23| The 1D-electron diffraction extracted from SAED for evaluating the domain size within the DPP-3D-COF film. The FWHM of the peak was measured as 0.21 nm^{-1}, which corresponds to a crystallite correlation length of 27.0 nm, according to the Scherrer equation $\xi_{hkl}=K\lambda/(B_{hkl}\cos\theta_{hkl})$. The crystallite correlation length relates to the average size of the region over which the repeating lattice is similarly aligned.’

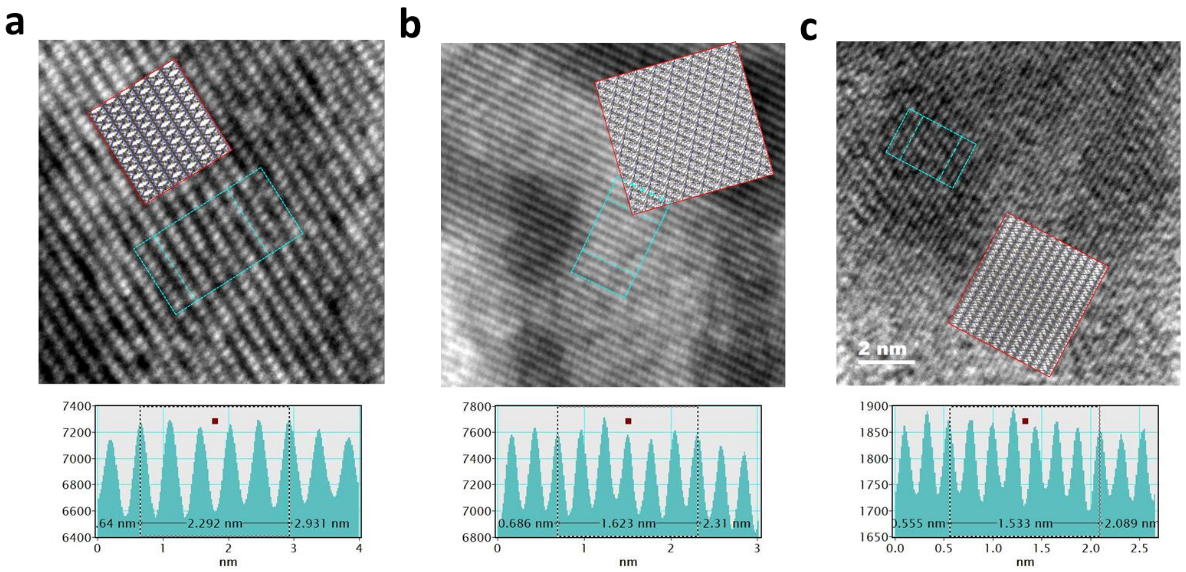

Supplementary Fig24| The distance measuring of lattice planes and matching with respective crystal structures. The relative intensities of the TEM images were extracted at the cyan dotted line within the cyan square and are shown below respective TEM image. The image in the red squares show structures of five-fold interpenetrated DPP-3D-COF in different directions that match with TEM patterns. The fivefold interpenetrated structure used are shown in Supplementary Fig. 26. **a**, The statistics of 5 chosen lattice gives an average lattice distance of 4.6 Å. **b**, The statistics of 6 chosen lattice gives an average lattice distance of 2.7 Å. **c**, The statistics of 7 chosen lattice gives an average lattice distance of 2.2 Å.

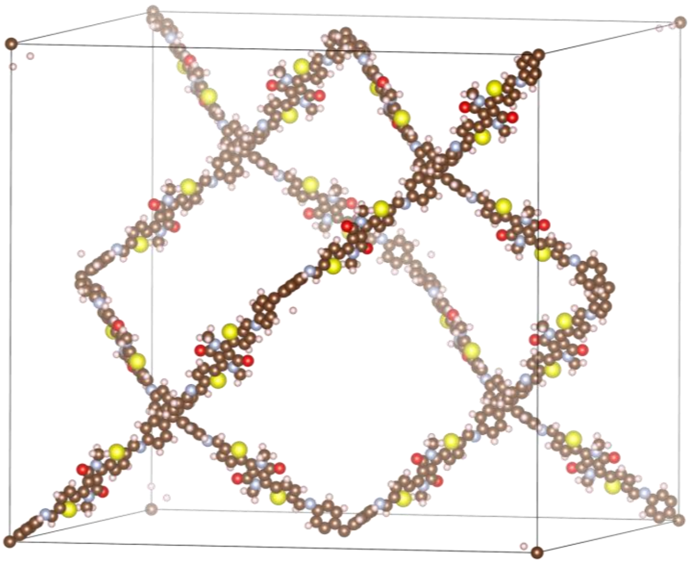

Supplementary Fig. 25| Calculated structure of a non-penetrated DPP-3D-COF. To simplify the calculation, the long alkyl chains were replaced by methyl groups.

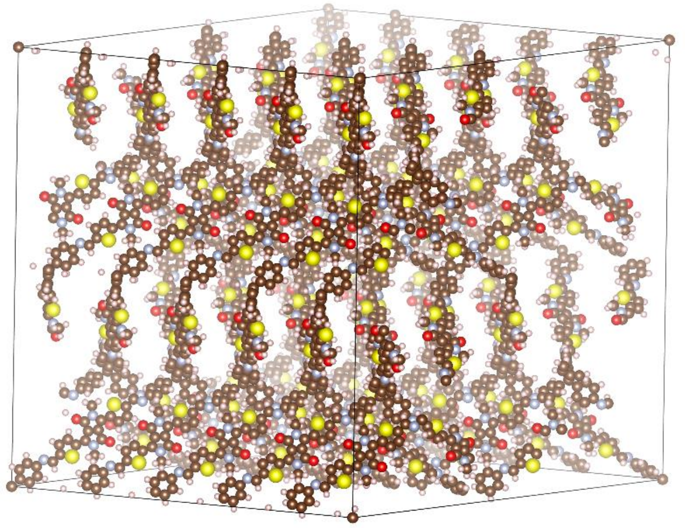

Supplementary Fig. 26| Calculated structure of a five-fold interpenetrated DPP-3D-COF in topology of *dia-c5*. To simplify the calculation, the long alkyl chains were replaced by methyl groups.

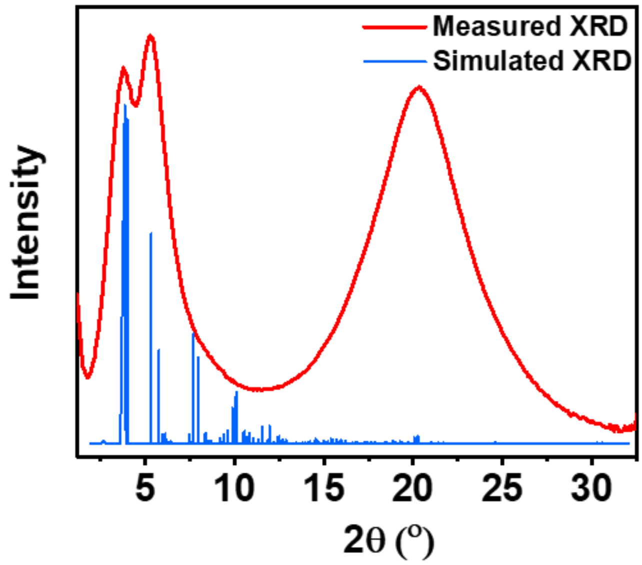

Supplementary Fig. 27| The GIXRD of a stack of DPP-3D-COF films. To obtain an acceptable signal-noise ratio, multiple batches of films were accumulated and piled on a SiO_2/Si chip for the GIXRD measurement. The simulated XRD are based on five-fold interpenetrated DPP-3D-COF structure with the large aliphatic side chains replaced by a methyl group.

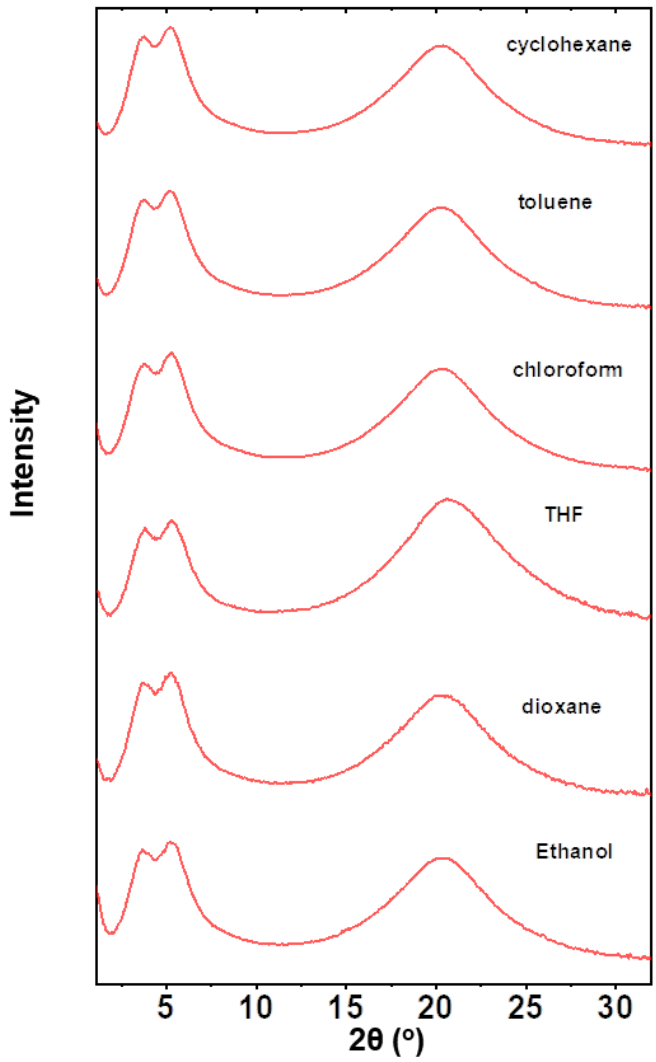

Supplementary Fig. 28| GIXRD spectra of DPP-3D-COF after treatment of different solvents. The prepared films were firstly immersed into respective solvents for 5 minutes and then moved out and dried for GIXRD measurements.

Supplementary Tab. 1| Atomic coordinates for the simulated non-penetrated DPP-3D-COF

(a = 60.92243, b = 66.60469, c = 61.64463, α = 91.9995, β = 87.5057, γ = 89.3348)

Atom	x	y	z
C1	4.00E-06	1	1
C2	0.22683	0.26532	0.25425
C3	0.21257	0.25315	0.23854
C4	0.23612	0.22699	0.21885
C5	0.22196	0.21599	0.206
C6	0.18327	0.23099	0.21265
C7	0.1834	0.21021	0.17298
C8	0.16516	0.19559	0.16653
C9	0.12999	0.18906	0.19064
C10	0.12006	0.17434	0.17696
C11	0.14746	0.16901	0.14182
C12	0.14354	0.15405	0.12227
C13	0.11261	0.14293	0.1348
C14	0.11926	0.12969	0.10768
C15	0.15606	0.13277	0.07708
C16	0.2053	0.15459	0.06369
C17	0.08821	0.11882	0.1201
C18	0.08406	0.1042	0.10042
C19	0.11168	0.09827	0.06556
C20	0.10132	0.08421	0.05165
C21	0.0655	0.07895	0.07523
C22	0.04652	0.06542	0.06851
C23	0.02642	0.11833	0.17869
C24	0.07577	0.13994	0.16536
C25	0.15943	0.25739	0.23225
C26	0.17404	0.26854	0.24439
C27	0.04615	0.04364	0.03007
C28	0.02815	0.01694	0.05613
C29	0.05024	0.05529	0.99364
C30	0.01398	0.00328	0.04587
C31	0.03526	0.04192	0.98394
C32	0.01655	0.01588	0.00982
C33	0.21264	0.24864	0.29655
C34	0.22807	0.25053	0.31546
C35	0.21539	0.23674	0.35364
C36	0.18632	0.22054	0.37442
C37	0.18901	0.19138	0.43262
C38	0.16888	0.18185	0.47272
C39	0.12925	0.19159	0.49341
C40	0.11802	0.17813	0.53168
C41	0.14881	0.15751	0.54161
C42	0.14419	0.14051	0.57941

C43	0.10932	0.14186	0.61118
C44	0.11662	0.1213	0.64331
C45	0.15793	0.10675	0.63062
C46	0.21346	0.11019	0.56963
C47	0.08174	0.12241	0.67508
C48	0.07711	0.10468	0.71284
C49	0.10788	0.08331	0.72276
C50	0.09662	0.06856	0.76097
C51	0.05699	0.07807	0.78164
C52	0.0368	0.06705	0.82167
C53	0.01247	0.15279	0.68485
C54	0.06801	0.15634	0.62388
C55	0.17096	0.21831	0.35573
C56	0.1844	0.23158	0.31748
C57	0.03939	0.03449	0.87981
C58	0.01048	0.05862	0.90149
C59	0.05466	0.99855	0.89765
C60	0.99803	0.04695	0.93968
C61	0.04148	0.98709	0.93592
C62	0.01358	0.01114	0.95771
C63	0.27081	0.25254	0.23737
C64	0.29048	0.21592	0.24921
C65	0.33009	0.20301	0.23445
C66	0.35157	0.22666	0.20684
C67	0.41262	0.19263	0.20629
C68	0.45377	0.18064	0.1856
C69	0.47417	0.18909	0.14705
C70	0.51373	0.17381	0.13517
C71	0.52498	0.15313	0.16436
C72	0.56425	0.13527	0.1589
C73	0.59643	0.13558	0.12461
C74	0.63014	0.11545	0.13074
C75	0.61804	0.10203	0.17069
C76	0.55594	0.10668	0.22557
C77	0.66232	0.11615	0.09645
C78	0.70161	0.0995	0.09099
C79	0.71289	0.08003	0.12018
C80	0.7525	0.06686	0.1083
C81	0.77289	0.07566	0.06974
C82	0.81407	0.06636	0.04904
C83	0.67062	0.14466	0.02977
C84	0.60853	0.14913	0.08466
C85	0.33221	0.26324	0.19481
C86	0.29257	0.27592	0.21033
C87	0.87524	0.03937	0.04838
C88	0.89625	0.02154	0.02051

C89	0.89508	0.04448	0.06049
C90	0.93588	0.00928	0.00553
C91	0.93472	0.03273	0.04473
C92	0.95602	0.01438	0.01738
C93	0.21079	0.30701	0.2447
C94	0.21368	0.32701	0.20859
C95	0.19983	0.36456	0.19857
C96	0.18188	0.38358	0.22489
C97	0.18161	0.44285	0.1867
C98	0.16267	0.48161	0.17999
C99	0.12697	0.49955	0.20362
C100	0.11654	0.53723	0.18952
C101	0.14399	0.54944	0.15444
C102	0.13969	0.5871	0.13445
C103	0.10862	0.61671	0.14669
C104	0.11507	0.64946	0.11919
C105	0.15181	0.63944	0.0886
C106	0.20126	0.58189	0.07567
C107	0.08412	0.67909	0.13153
C108	0.08001	0.71674	0.11161
C109	0.10716	0.7288	0.0762
C110	0.09711	0.76648	0.06223
C111	0.06209	0.7846	0.08637
C112	0.04384	0.82345	0.07981
C113	0.02258	0.68434	0.1903
C114	0.07192	0.62674	0.17732
C115	0.1776	0.36378	0.26133
C116	0.19222	0.32625	0.27081
C117	0.04376	0.88235	0.04005
C118	0.00494	0.89924	0.04722
C119	0.06773	0.90404	0.02017
C120	0.99076	0.93666	0.03473
C121	0.0531	0.94136	0.00841
C122	0.0144	0.9585	0.01491
N1	0.16678	0.21884	0.20326
N2	0.16925	0.14779	0.08776
N3	0.06249	0.12509	0.15461
N4	0.06251	0.05737	0.03802
N5	0.16992	0.20905	0.41299
N6	0.17294	0.11951	0.59149
N7	0.05299	0.14351	0.663
N8	0.05579	0.04529	0.84123
N9	0.39135	0.21484	0.18907
N10	0.57763	0.11501	0.18614
N11	0.64893	0.13626	0.06921
N12	0.83545	0.05011	0.06625

N13	0.16567	0.42113	0.21717
N14	0.16523	0.60113	0.09969
N15	0.05859	0.66507	0.16629
N16	0.06023	0.84486	0.04931
S1	0.1851	0.18303	0.12709
S2	0.04561	0.09168	0.11456
S3	0.19128	0.15598	0.50188
S4	0.03462	0.10549	0.75252
S5	0.48481	0.15352	0.20619
S6	0.74179	0.10029	0.04916
S7	0.18238	0.51255	0.14038
S8	0.04244	0.75379	0.12622
O1	0.17422	0.12497	0.04758
O2	0.05766	0.14763	0.19491
O3	0.17816	0.08758	0.64742
O4	0.04778	0.1755	0.60707
O5	0.63613	0.08355	0.18961
O6	0.59043	0.16764	0.06573
O7	0.16974	0.65766	0.05878
O8	0.05393	0.60853	0.20708
H1	0.26641	0.21457	0.21315
H2	0.24112	0.19479	0.19154
H3	0.21164	0.21373	0.15056
H4	0.11212	0.19495	0.2175
H5	0.09346	0.1675	0.1921
H6	0.20222	0.18354	0.05493
H7	0.21764	0.14621	0.03885
H8	0.22553	0.13891	0.07657
H9	0.13871	0.10426	0.05074
H10	0.11927	0.078	0.02494
H11	0.01762	0.06332	0.09043
H12	0.00602	0.13438	0.16599
H13	0.01433	0.1263	0.20372
H14	0.02942	0.08945	0.18709
H15	0.12932	0.26966	0.23734
H16	0.15479	0.28949	0.25919
H17	0.02603	0.00605	0.08434
H18	0.06458	0.07572	0.97292
H19	7.51E-04	0.98201	0.0666
H20	0.03898	0.05187	0.95525
H21	0.25062	0.26317	0.30002
H22	0.22748	0.2395	0.36745
H23	0.2209	0.18205	0.42036
H24	0.10915	0.20809	0.48073
H25	0.08807	0.18302	0.55214
H26	0.22315	0.09437	0.55121

H27	0.22717	0.09344	0.58871
H28	0.22318	0.1343	0.55276
H29	0.13784	0.07872	0.70232
H30	0.1167	0.05126	0.77363
H31	0.00492	0.0778	0.83391
H32	0.00271	0.1287	0.70134
H33	0.99878	0.16992	0.66577
H34	0.00282	0.16821	0.70362
H35	0.14873	0.2053	0.37143
H36	0.17206	0.2287	0.30401
H37	0.99841	0.08698	0.88841
H38	0.0767	0.97928	0.88126
H39	0.97568	0.06643	0.95582
H40	0.05381	0.95872	0.9487
H41	0.27452	0.19683	0.27067
H42	0.34437	0.17418	0.24386
H43	0.40101	0.18231	0.23715
H44	0.46038	0.20603	0.12816
H45	0.53419	0.17757	0.10588
H46	0.53845	0.13123	0.23546
H47	0.57621	0.0899	0.23819
H48	0.53718	0.09126	0.23524
H49	0.69244	0.07575	0.14947
H50	0.76631	0.05123	0.12718
H51	0.82562	0.07469	0.01817
H52	0.68878	0.16074	0.02006
H53	0.65034	0.16084	0.01715
H54	0.68874	0.12011	0.01993
H55	0.3485	0.28208	0.17369
H56	0.27864	0.3048	0.20073
H57	0.88158	0.01647	0.01104
H58	0.87915	0.05871	0.08182
H59	0.95148	0.99549	0.98384
H60	0.94903	0.03805	0.05438
H61	0.22687	0.31294	0.18764
H62	0.20213	0.37905	0.17033
H63	0.21043	0.4334	0.16467
H64	0.10913	0.48538	0.23051
H65	0.08958	0.55567	0.20433
H66	0.22177	0.56584	0.08824
H67	0.21322	0.60199	0.05044
H68	0.19822	0.56381	0.06763
H69	0.13368	0.71025	0.06108
H70	0.1148	0.78056	0.03513
H71	0.01582	0.83326	0.1024
H72	0.0257	0.7021	0.19867

H73	0.01038	0.66426	0.21536
H74	0.00223	0.70074	0.17755
H75	0.16332	0.37813	0.28225
H76	0.18832	0.31193	0.29952
H77	0.98567	0.88309	0.06187
H78	0.09796	0.8914	0.01464
H79	0.96035	0.94875	0.04088
H80	0.07244	0.95752	0.99348

Supplementary Tab. 2. Atomic coordinates for the simulated *dia-c5* interpenetrated DPP-3D-COF

(a = 12.1845, b = 31.1574, c = 33.7854, α = 89.498, β = 80.277, γ = 98.771)

Atoms	x	y	z
C1	0.98664	0.99955	0.99995
C2	0.77219	0.49171	0.51887
C3	0.67428	0.46527	0.54885
C4	0.56027	0.46265	0.54498
C5	0.47454	0.4375	0.57199
C6	0.50081	0.41381	0.60403
C7	0.31961	0.39317	0.64357
C8	0.23816	0.3603	0.66826
C9	0.25569	0.31859	0.67933
C10	0.1606	0.29395	0.70293
C11	0.0666	0.31603	0.71067
C12	0.95911	0.29713	0.73415
C13	0.93246	0.25509	0.75254
C14	0.81801	0.24851	0.77301
C15	0.77225	0.28839	0.76681
C16	0.84678	0.35945	0.73096
C17	0.7916	0.20657	0.79164
C18	0.68456	0.18781	0.81547
C19	0.58979	0.2095	0.82271
C20	0.49553	0.18507	0.84699
C21	0.51469	0.14401	0.85921
C22	0.43494	0.11149	0.88492
C23	0.90411	0.14429	0.79485
C24	0.97831	0.21526	0.75882
C25	0.61499	0.41637	0.60827
C26	0.69946	0.44213	0.58152
C27	0.25392	0.08934	0.92373
C28	0.23399	0.04464	0.91567
C29	0.18363	0.10507	0.95608
C30	0.14808	0.01681	0.94009
C31	0.09948	0.07673	0.98075
C32	0.08029	0.03199	0.97358

C33	0.83485	0.46083	0.49076
C34	0.91268	0.47815	0.45642
C35	0.97317	0.45169	0.43092
C36	0.95783	0.40641	0.43922
C37	0.04762	0.37994	0.37833
C38	0.11924	0.35029	0.35835
C39	0.17089	0.32039	0.37729
C40	0.23406	0.2957	0.35025
C41	0.23281	0.30587	0.30953
C42	0.29059	0.28425	0.27635
C43	0.3551	0.25073	0.27944
C44	0.39709	0.23747	0.24003
C45	0.35665	0.26423	0.21141
C46	0.23956	0.32321	0.21686
C47	0.46085	0.20371	0.24313
C48	0.5163	0.18133	0.21001
C49	0.51272	0.19073	0.16931
C50	0.57184	0.16473	0.14236
C51	0.62275	0.13461	0.16133
C52	0.68962	0.1034	0.14148
C53	0.51202	0.16481	0.30264
C54	0.3953	0.2239	0.30807
C55	0.87971	0.38881	0.47327
C56	0.81822	0.41553	0.49807
C57	0.76893	0.07343	0.08075
C58	0.87745	0.06865	0.08798
C59	0.72992	0.05277	0.04764
C60	0.94457	0.04453	0.06223
C61	0.79712	0.02811	0.02256
C62	0.90679	0.02427	0.02866
C63	0.72718	0.5229	0.49177
C64	0.6725	0.50595	0.46027
C65	0.62913	0.53265	0.43541
C66	0.63869	0.57778	0.44154
C67	0.59601	0.60479	0.38105
C68	0.53912	0.63395	0.36059
C69	0.46922	0.66281	0.37874
C70	0.42728	0.68709	0.35106
C71	0.46408	0.67765	0.31061
C72	0.43339	0.69907	0.27681
C73	0.36365	0.73157	0.27891
C74	0.35534	0.74514	0.23908
C75	0.42281	0.71961	0.21123
C76	0.53936	0.66217	0.21843
C77	0.28676	0.77802	0.24118
C78	0.25972	0.80067	0.20735

C79	0.30017	0.79247	0.16687
C80	0.26462	0.81891	0.13915
C81	0.19572	0.84809	0.15732
C82	0.14691	0.87998	0.13684
C83	0.18058	0.81483	0.29955
C84	0.29656	0.75721	0.30676
C85	0.693	0.59499	0.47293
C86	0.73796	0.56804	0.49705
C87	0.12514	0.91417	0.07633
C88	0.00904	0.91735	0.08319
C89	0.19662	0.93911	0.04439
C90	0.96695	0.94471	0.05854
C91	0.15375	0.96701	0.02049
C92	0.03796	0.96995	0.02655
C93	0.85255	0.51735	0.54447
C94	0.80712	0.54024	0.57768
C95	0.87586	0.56393	0.60155
C96	0.99393	0.56501	0.59318
C97	0.05687	0.62401	0.63164
C98	0.13411	0.64383	0.65729
C99	0.22315	0.62607	0.66935
C100	0.28346	0.65333	0.69389
C101	0.24226	0.69297	0.70153
C102	0.29094	0.72633	0.72582
C103	0.38545	0.72488	0.74465
C104	0.40769	0.76409	0.76569
C105	0.32256	0.7908	0.75955
C106	0.16058	0.78271	0.72302
C107	0.50264	0.76277	0.7843
C108	0.55173	0.79631	0.80833
C109	0.50878	0.83551	0.81659
C110	0.56989	0.86315	0.84061
C111	0.66165	0.84625	0.85149
C112	0.74026	0.86685	0.8763
C113	0.63314	0.70647	0.78707
C114	0.47074	0.69821	0.75071
C115	0.03957	0.54093	0.56102
C116	0.97006	0.51803	0.53692
C117	0.79758	0.92566	0.91614
C118	0.83098	0.90374	0.94779
C119	0.82701	0.97133	0.91205
C120	0.89156	0.92697	0.97446
C121	0.88902	0.99401	0.93844
C122	0.92124	0.97245	0.97064
N123	0.41974	0.38517	0.62991
N124	0.86253	0.31659	0.74294

N125	0.88823	0.18713	0.78285
N126	0.33532	0.11943	0.89942
N127	0.02269	0.37853	0.41703
N128	0.29258	0.29201	0.23552
N129	0.45915	0.19605	0.28396
N130	0.70196	0.10063	0.10294
N131	0.58969	0.60573	0.41954
N132	0.46771	0.69219	0.23618
N133	0.25199	0.78475	0.28181
N134	0.17117	0.88511	0.09824
N135	0.06721	0.58635	0.61711
N136	0.25435	0.76591	0.73503
N137	0.53931	0.72321	0.77507
N138	0.72937	0.90464	0.89041
S139	0.10208	0.36768	0.68776
S140	0.65098	0.13685	0.83977
S141	0.1515	0.34681	0.30679
S142	0.59528	0.13966	0.21281
S143	0.55054	0.63787	0.30896
S144	0.17677	0.84163	0.209
S145	0.12781	0.69447	0.6772
S146	0.6691	0.79578	0.8313
O147	0.67852	0.29875	0.77815
O148	0.07195	0.20485	0.74737
O149	0.3698	0.26529	0.17437
O150	0.38213	0.22283	0.3451
O151	0.44225	0.71923	0.17421
O152	0.27717	0.75762	0.34379
O153	0.3057	0.82695	0.77143
O154	0.48737	0.66201	0.73894
H155	0.53616	0.48129	0.5194
H156	0.38284	0.43603	0.56821
H157	0.29485	0.42582	0.63636
H158	0.34101	0.30783	0.66911
H159	0.16456	0.25958	0.71384
H160	0.80323	0.37572	0.758
H161	0.7924	0.35682	0.70648
H162	0.93225	0.37968	0.71894
H163	0.59493	0.24357	0.80923
H164	0.41385	0.19914	0.85489
H165	0.4605	0.07901	0.89225
H166	0.85805	0.11904	0.77671
H167	0.86894	0.138	0.82821
H168	0.99791	0.14197	0.78912
H169	0.6385	0.39729	0.6337
H170	0.79044	0.44433	0.5864

H171	0.28814	0.03128	0.88919
H172	0.19607	0.1413	0.96199
H173	0.92729	0.51455	0.44899
H174	0.03504	0.46658	0.40336
H175	0.01415	0.40412	0.35976
H176	0.1592	0.31804	0.41148
H177	0.27999	0.27037	0.36205
H178	0.19028	0.34144	0.24109
H179	0.17959	0.30559	0.19766
H180	0.30688	0.34725	0.19707
H181	0.46486	0.21731	0.16115
H182	0.57494	0.16928	0.10865
H183	0.73054	0.08167	0.16001
H184	0.47994	0.13076	0.29327
H185	0.60731	0.17236	0.29272
H186	0.49062	0.16659	0.3367
H187	0.86633	0.35243	0.48074
H188	0.7535	0.40002	0.52467
H189	0.66295	0.46965	0.45467
H190	0.58593	0.51805	0.41012
H191	0.64595	0.5812	0.36304
H192	0.45165	0.66472	0.41286
H193	0.3694	0.71155	0.3622
H194	0.48543	0.63266	0.20806
H195	0.58832	0.65131	0.24163
H196	0.601	0.67882	0.19157
H197	0.35605	0.76642	0.15946
H198	0.29209	0.81535	0.10553
H199	0.08738	0.90034	0.15493
H200	0.19173	0.81975	0.33211
H201	0.08883	0.80123	0.29851
H202	0.2047	0.8473	0.28205
H203	0.70048	0.63121	0.47878
H204	0.78414	0.58324	0.5214
H205	0.94964	0.89755	0.10887
H206	0.29027	0.93659	0.0379
H207	0.71213	0.53962	0.58552
H208	0.83683	0.58248	0.62785
H209	0.98712	0.64175	0.62438
H210	0.24024	0.59258	0.65913
H211	0.35778	0.64249	0.70552
H212	0.10939	0.79607	0.75023
H213	0.10475	0.75551	0.71017
H214	0.19346	0.80982	0.69917
H215	0.43154	0.84208	0.80404
H216	0.54309	0.89574	0.84937

H217	0.81123	0.84959	0.88323
H218	0.62683	0.67042	0.78056
H219	0.63138	0.71099	0.82061
H220	0.7154	0.72475	0.76954
H221	0.13408	0.54004	0.55451
H222	0.01016	0.49964	0.5107
H223	0.8	0.98985	0.88703
H224	0.91025	0.08449	0.11488
H225	0.64258	0.05636	0.04147
H226	0.80856	0.86689	0.95169
H227	0.91801	0.90907	0
H228	0.13116	0.98088	0.93324
H229	0.03218	0.04082	0.06805
H230	0.87319	0.94709	0.064
H231	0.21281	0.98797	0.99549
H232	0.91486	0.03072	0.93433
H233	0.0447	0.08969	0.0073
H234	0.76349	0.01061	0.99655

Supplementary Reference

1. Ma, T. *et al.* Observation of Interpenetration Isomerism in Covalent Organic Frameworks. *J. Am. Chem. Soc.* **140**, 6763-6766 (2018).
